# Heterologous expression of the avirulence gene *ACE1* from the fungal rice pathogen *Magnaporthe oryzae* †
†Electronic supplementary information (ESI) available. See DOI: 10.1039/c4sc03707c



**DOI:** 10.1039/c4sc03707c

**Published:** 2015-06-01

**Authors:** Zhongshu Song, Walid Bakeer, James W. Marshall, Ahmed A. Yakasai, Rozida Mohd Khalid, Jerome Collemare, Elizabeth Skellam, Didier Tharreau, Marc-Henri Lebrun, Colin M. Lazarus, Andrew M. Bailey, Thomas J. Simpson, Russell J. Cox

**Affiliations:** a School of Chemistry , University of Bristol , Cantock's Close , Bristol , BS8 1TS , UK; b Microbiology Department , Faculty of Pharmacy , Beni Suef University , Egypt; c UMR1345 , IRHS-INRA , 49071 Beaucouzé Cedex , France; d Institute for Organic Chemistry , Leibniz University of Hannover , Schneiderberg 1B , 30167 , Hannover , Germany . Email: russell.cox@oci.uni-hannover.de; e UMR BGPI , CIRAD , Campus International de Baillarguet , 34398 Montpellier Cedex 5 , France; f UR 1290 BIOGER-CPP , INRA , Campus AgroParisTech , 78850 Thiverval-Grignon , France; g UMR 5240 MAP , CNRS , UCB , INSA , Bayer CropScience , 69263 Lyon Cedex 09 , France; h School of Biological Sciences , University of Bristol , 24 Tyndall Avenue , Bristol BS8 1TQ , UK

## Abstract

The *ACE1* and *RAP1* genes from the avirulence signalling gene cluster of the rice blast fungus *Magnaporthe oryzae* were expressed in *Aspergillus oryzae* and *M. oryzae* itself. Expression of *ACE1* alone produced a polyenyl pyrone (magnaporthepyrone), which is regioselectively epoxidised and hydrolysed to give different diols, **6** and **7**, in the two host organisms. Analysis of the three introns present in *ACE1* determined that *A. oryzae* does not process intron 2 correctly, while *M. oryzae* processes all introns correctly in both appressoria and mycelia. Co-expression of *ACE1* and *RAP1* in *A. oryzae* produced an amide **8** which is similar to the PKS-NRPS derived backbone of the cytochalasans. Biological testing on rice leaves showed that neither the diols **6** and **7**, nor amide **8** was responsible for the observed *ACE1* mediated avirulence, however, gene cluster analysis suggests that the true avirulence signalling compound may be a tyrosine-derived cytochalasan compound.

## Abstract 2

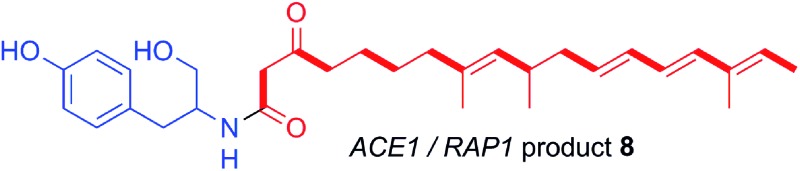
Heterologous expression of key components of the *Magnaporthe grisea ACE1* gene cluster produces a potential precursor of cryptic avirulence signalling compounds that induce resistance to *M. grisea* in rice.

## Introduction

The rice blast fungus *Magnaporthe oryzae*, which causes significant reductions in rice yields,^1^ is a well-known model for studying plant fungal interactions and pathogenicity.^2^ Rice resistance receptors (R) recognize *M. oryzae* secreted avirulence signals (AVR), allowing the rice plant to mount an effective defence response, killing or containing the fungus within the initial infected cell. Genetic and molecular studies have conclusively linked the avirulent phenotype of strain Guy11 on rice cultivars carrying the resistance gene *Pi33*, to the presence of the *ACE1* gene (Avirulence Conferring Enzyme).^3,4^ Unlike the majority of known *AVR* genes which encode small secreted proteins acting directly on the host plant,^5^ *ACE1* encodes a cytoplasmic biosynthetic protein responsible for the production of a low molecular weight compound.^4^

**Scheme 1:**
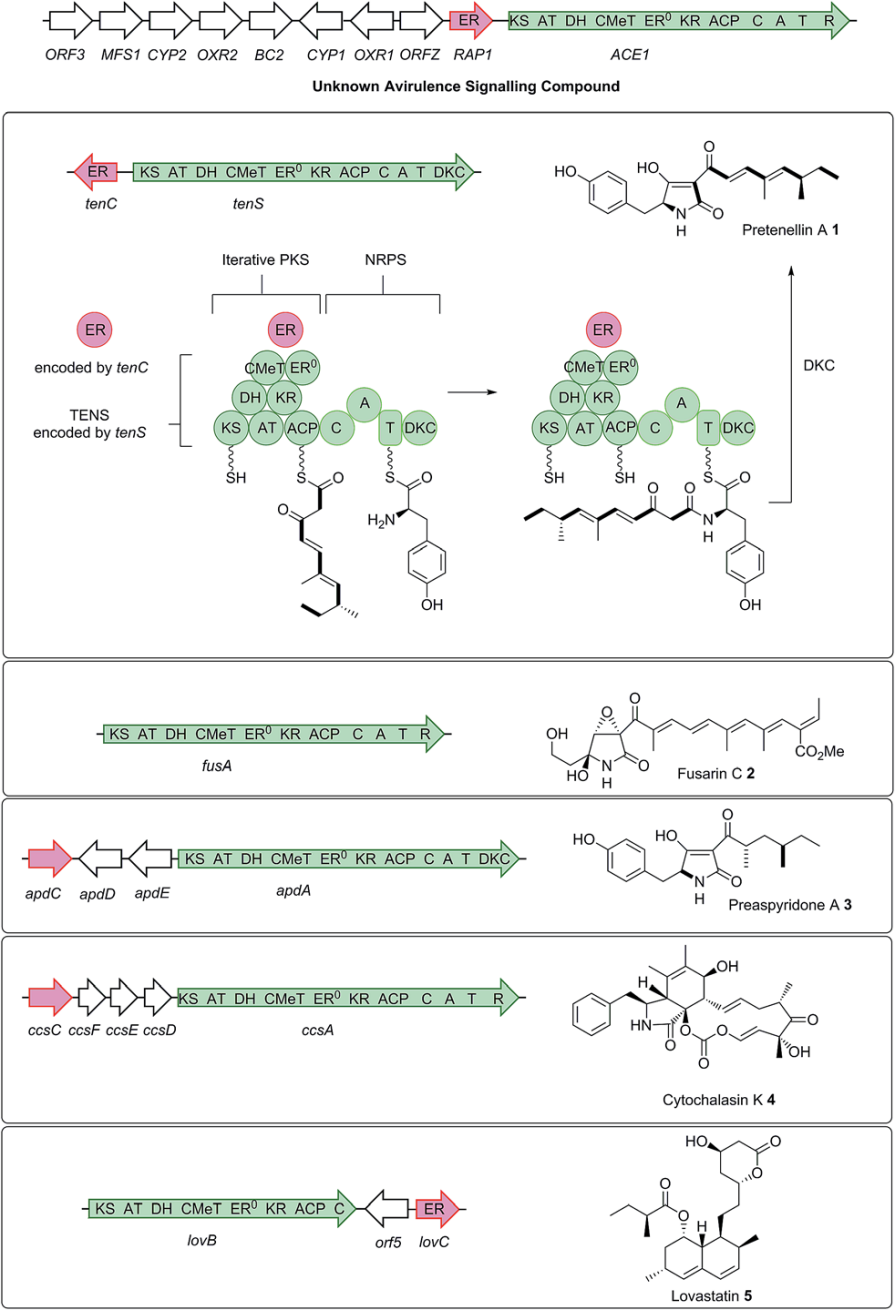
Relationship between biosynthetic gene clusters and the structures of known compounds in various fungi (only partial gene clusters are shown). The *ACE1* gene cluster (MGG_08381 – MGG_08391, MGG_12447)^6^ is shown and the *ACE1*/*RAP1* gene pair which show significant homology to other genes known to produce bioactive compounds are highlighted. The top panel shows key chemical steps catalysed by the fungal PKS-NRPS TENS, which synthesises pretenellin A **1** in partnership with the *trans*-acting ER TENC. KS, β-ketoacyl synthase; AT, acyl transferase, DH, dehydratase; ER, enoyl reductase; ER^O^, defective enoyl reductase; *C*MeT, *C*-methyl transferase; KR, β-ketoacyl reductase; ACP, acyl carrier protein; C, condensation; A, adenylation; T, thiolation; DKC, Dieckmann cyclase; R, thiolester reductase.

*ACE1* belongs to an infection-specific secondary metabolite gene cluster (Scheme 1),^6^ expressed only during appressorium-mediated penetration, and not at any other stage of the *M. oryzae* life cycle.^7^ The 12.4 kb *ACE1* gene encodes an enzyme consisting of a fungal highly-reducing polyketide synthase (hrPKS)^8^ fused to a single module of a nonribosomal peptide synthetase (NRPS).^4^ Similar fungal synthetases have been investigated genetically and biochemically and are known to produce acyl tetramic acids or pyrrolidones including pretenellin A **1**,^9^ prefusarin C **2** (ref. 10) and preaspyridone A **3** (ref. 11 and 12) as well as other potent bioactive compounds including cytochalasin K **4** (ref. 13 and 14) and the important cholesterol biosynthesis inhibitor lovastatin **5** (ref. 15) (Scheme 1). It appears likely, therefore, that *ACE1* encodes a biosynthetic protein which makes a small molecule consisting of a polyketide fused to an amino acid. Because *ACE1* is under very tight temporal and cell type-specific control in *M. oryzae*, it has not yet been possible to identify or purify the avirulence compound. Knowledge of the *ACE1* metabolite structure would be highly useful for investigating avirulence signalling and developing novel resistant cultivars. The research of our group and that of others has focused on the heterologous expression of other fungal PKS-NRPS genes in *Aspergillus oryzae* from gene clusters of *known* function.^12,16–19^ Here we set out to use heterologous expression of genes from a *cryptic pathway* to determine its unknown chemical product, since knowledge of the *ACE1* compound will enhance efforts to design new compounds of high potential utility in crop protection.

## Results

**Fig. 1:**
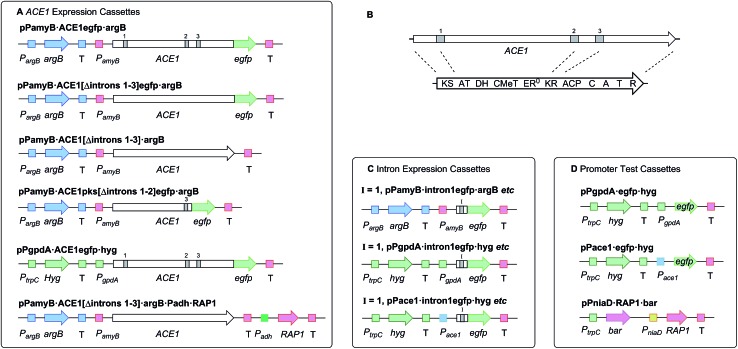
Diagrammatic representation of fungal expression systems used in this work (not to scale). Introns indicated by grey bars. PKS-NRPS functional domains as described in the legend to Scheme 1. P = promoter, T = terminator. (A) cassettes used for expression of *ACE1* and *RAP1*; (B) relative positions of *ACE1* introns in cDNA and mRNA; (C) cassettes used for testing intron splicing, *I* = 1/2/3; (D) cassettes used for promoter testing and coexpression of *RAP1*.

In initial experiments a construct known to express *ACE1* in *M. oryzae*, containing *ACE1* fused to *eGFP* at its 3′ terminus (pPACE1·ACE1eGFP·hyg),^4^ was used to construct the vector pPamyB·ACE1eGFP·argB (Fig. 1A) in which the *ACE1-eGFP* gene fusion was located downstream of *P*_*amyB*_.

This plasmid was then used to transform *A. oryzae* M-2-3. Transformants were selected on minimal medium and 100 colonies were selected and grown further on both minimal medium and minimal medium plus maltose. Four transformants showed weak fluorescence and these were picked together with 10 non-fluorescent transformants and grown in liquid medium (see ESI†). The resultant organic extracts were examined by LCMS but no significant chemical differences were observed between extracts from any of the transformants and the untransformed *A. oryzae* M-2-3 strain (data not shown).

### Intron processing

Since *A. oryzae* is increasingly being used as a heterologous host for studying gene expression, we wanted to determine why our initial attempt at expressing *ACE1* had failed. The *ACE1* gene contains three introns located between exons corresponding to PKS domains (Fig. 1B). As there is a possibility that *A. oryzae* may not always splice heterologously expressed genes the same way as the host organism,^18,20^ we explored how the introns of *ACE1* are processed by *A. oryzae* and *M. oryzae*.

**Fig. 2:**
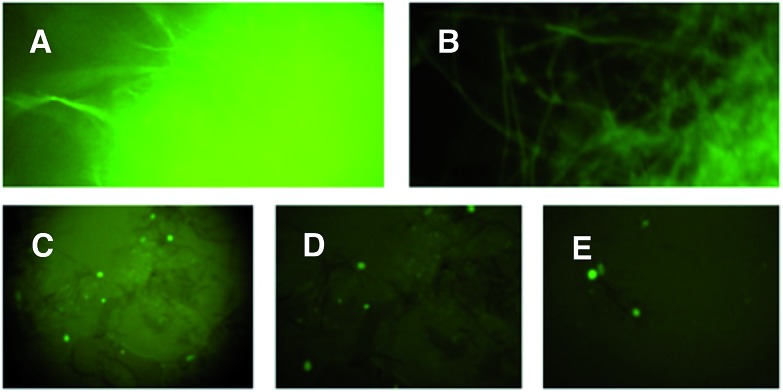
UV-stimulated green fluorescence of *intron-eGFP* clones. (A) *A. oryzae* M-2-3 pPamyB·intron3eGFP·argB; (B) *M. oryzae* Guy11 pPgpdA·intron2eGFP·hyg; (C) *M. oryzae* Guy11 pPACE1·intron1eGFP·hyg; (D) *M. oryzae* Guy11 pPACE1·intron2eGFP·hyg; (E) *M. oryzae* Guy11 pPACE1·intron3eGFP·hyg. A and B mycelia grown in liquid; C, D and E appressoria grown on cellophane.

Each *ACE1* intron was amplified with *ca.* 20 bp of flanking 5′ and 3′ exon sequences and cloned as in-frame fusions with *eGFP* downstream of either the native *ACE1* promoter (*P*_*Ace1*_), the strong constitutive promoter *P*_*gpdA*_ from *Aspergillus nidulans* or the inducible promoter *P*_*amyB*_ from *A. oryzae*. A total of nine plasmids were constructed (Fig. 1C). These constructs were transformed into either *A. oryzae* or *M. oryzae*, along with positive and negative controls, selected on the appropriate selection media and observed for fluorescence (Table 1 and Fig. 2). RT-PCR was performed for each intron construct using primers located either side of the introns and the products were sequenced (see ESI† for details).

**Table 1:** Results of the *ACE1* intron processing constructs. Conditions: M, mycelia grown *in vitro*; A, apressoria grown on cellophane

Construct name	Expression host	Conditions	Fluorescent transformants
pPamyB·intron1·eGFP·argB	*A. oryzae*	M	80%
pPamyB·intron2·eGFP·argB	*A. oryzae*	M	0%
pPamyB·intron3·eGFP·argB	*A. oryzae*	M	90% (Fig. 2A)
Empty vector	*A. oryzae*	M	0%
eGFP	*A. oryzae*	M	87%
pPgpdA·intron1eGFP·hyg	*M. oryzae*	M, A	>70%
pPgpdA·intron2eGFP·hyg	*M. oryzae*	M, A	>70% (Fig. 2B)
pPgpdA·intron3eGFP·hyg	*M. oryzae*	M, A	>70%
Empty vector	*M. oryzae*	M, A	0%
eGFP	*M. oryzae*	A	>70%
pPACE1·intron1eGFP·hyg	*M. oryzae*	A	>60% (Fig. 2C)
pPACE1·intron2eGFP·hyg	*M. oryzae*	A	>60% (Fig. 2D)
pPACE1·intron3eGFP·hyg	*M. oryzae*	A	>60% (Fig. 2E)
Empty vector	*M. oryzae*	M	0%
eGFP	*M. oryzae*	M, A	>60%

The results demonstrated that introns 1 and 3 are spliced identically in *A. oryzae* and *M. oryzae* whereas incorrect splicing of intron 2 by *A. oryzae* results in a frameshift and premature stop codon (Fig. S1†).

### Heterologous expression of *ACE1*

**Fig. 3:**
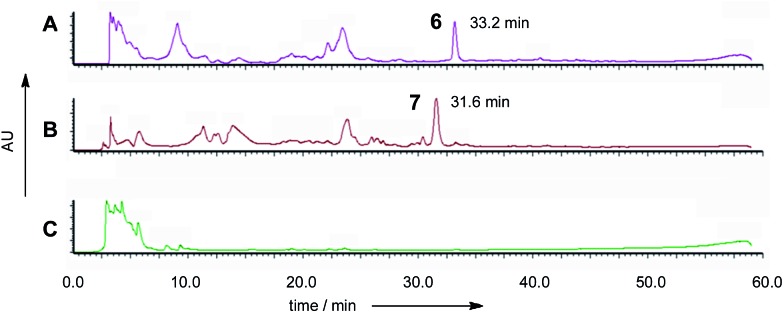
Diode array chromatograms of culture extracts from: (A) *A. oryzae* M-2-3 pPamyB·ACE1[Δintrons1–3]eGFP·argB; (B) *M. oryzae* pPgpdA·ACE1[Δintrons1–2]eGFP·hyg; (C) *A. oryzae* M-2-3 empty vector transformant.

Based on results from the intron processing experiments, we created two modified expression plasmids for further attempts to produce the *ACE1* compound (Fig. 1A). The first consisted of the entire *ACE1* gene lacking all introns fused to *eGFP* (pPamyB·ACE1[Δintrons1–3]eGFP·argB); the second consisted only of the PKS encoding portion of *ACE1* – also fused to *eGFP*, and containing only intron-3 (pAmyB·ACE1pks[Δintrons1–2]eGFP·argB, see ESI† for construction details). Controls included empty vector and *eGFP* alone (pPgpdA·eGFP·hyg, Fig. 1D). These plasmids were used to transform *A. oryzae* M-2-3 and selection was achieved on minimal medium supplemented with hygromycin where appropriate. On malt extract agar (MEA) the transformants containing the *ACE1* constructs produced a bright yellow colour, while the controls were colourless. Twenty colonies were selected for each construct, grown in liquid medium and extracted as described above. All transformants tested clearly produced a new peak compared to the controls (Fig. 3A) in the neutral organic extract, and in lesser amounts, in the acidic and mycelial extracts.

The new compounds appeared to be identical for the two *ACE1* expression constructs (RT = 33.2 min; uv max = 214, 272, 361 nm; *m*/*z* = 315 [M]Na^+^, 293 [M]H^+^, 275 [M – H_2_O]H^+^). Both compounds were purified and shown to be identical by NMR and HRMS (*m*/*z* 291.1238 Da [M – H]^–^, consistent with a molecular formula of C_16_H_20_O_5_).

^1^H NMR analysis (see ESI†) showed that the sample consisted of a 1 : 1 mixture of diastereomers each of which contained three methyl groups, a CHX, five contiguous olefinic protons, and two sharp doublets (*J* = 2.0 Hz) in the olefinic region. COSY correlations were used to determine that the olefinic protons were part of a distinct spin-system with an olefinic methyl group, connected by a vicinal coupling, visible only as a slight broadening of the terminal olefin doublet in the 1D ^1^H NMR. The COSY also showed that the CHX (which appeared as a complex multiplet) and one of the methyl groups (itself appearing as a pair of doublets) formed another distinct coupling system. An HMBC spectrum determined that the lone methyl group and olefins were all part of the same chain with a formula of C_11_H_17_O_2_, leaving C_5_H_3_O_3_ unaccounted for. The characteristic ^13^C NMR resonance of *δ* 163.6 ppm revealed that an ester group was present in the structure accounting for a further CO_2_, and leaving C_4_H_3_O unassigned.

The remaining two unassigned protons showed HMBC correlations to the ester carbonyl and two other quaternary carbons, the chemical shifts of which (*δ* 162.4 ppm and *δ* 162.6 ppm) were consistent with enolic carbons. Although these protons were coupled, they did not appear to be on adjacent carbons. An IR spectrum showed a characteristic absorbance at 1685 cm^–1^ suggesting that the ester group was present as part of a pyrone. Thus the structure was determined as the novel pyrone **6** to which we have assigned the name 12,13-dihydroxymagnaporthepyrone.

**Fig. 4:**
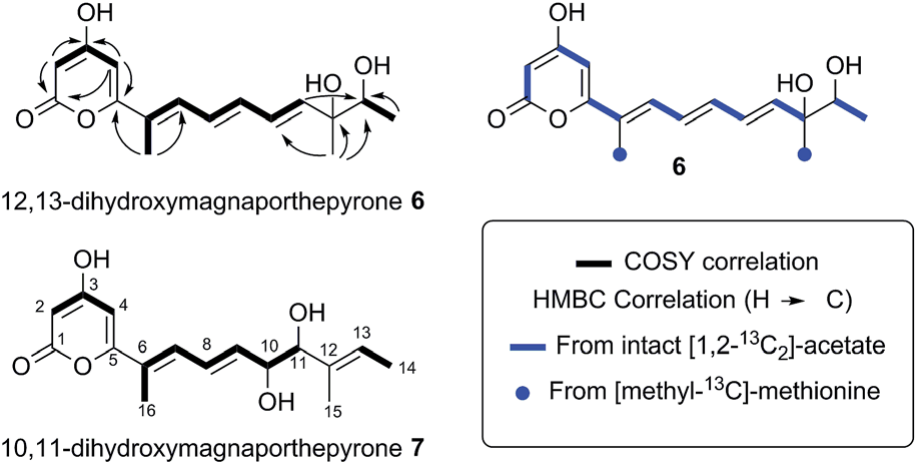
Structures and biosynthetic origin of the pyrones obtained by expressing *ACE1* in *A. oryzae* M-2-3 and *M. oryzae*.

The polyketide origin of **6** was confirmed by feeding [1,2-^13^C_2_]-acetate and [methyl-^13^C]-methionine to *A. oryzae* pPamyB·ACE1[Δintrons1–3]eGFP·argB. Pyrone **6** isolated after the labelled acetate feed showed the presence of distinctive doublets in the ^13^C NMR indicative of the incorporation of intact ^13^C_2_ units (Fig. 4 and ESI†). Matching of the ^2^ *J*_CC_ values showed intact acetate incorporations consistent with the heptaketide origin of **6**. The origin of both methyls from methionine was confirmed by the observation of a 9.4% incorporation of label at C-15 and C-16.

Thus **6** consists entirely of a polyketide and does not contain the expected amino acid component. To rule out NRPS perturbance by the C-terminal eGFP fusion we modified the expression system to remove the 3′-*eGFP* gene to create pPamyB·ACE1[Δintrons1–3]·argB and transformed *A. oryzae* M-2-3. Selected transformants were grown in liquid medium and secondary metabolites detected using LCMS; 12,13-dihydroxymagnaporthepyrone **6** was the only new compound detected.

To overcome the possibility that *A. oryzae* lacks the specific amino acid required by the ACE1 NRPS in *M. oryzae* we constructed plasmids for homologous expression of *ACE1* in *M. oryzae* Guy11 using the *P*_*gpdA*_ constitutive promoter and hygromycin selection. We thus constructed an *E. coli* shuttle vector based on the pUC18 vector with a Gateway cassette downstream from *P_gpdA_* carrying a hygromycin resistance marker. The *ACE1 eGFP* construct was transferred into this vector by Gateway LR recombination *in vitro* to give pPgpdA·ACE1eGFP·hyg which was used to transform *M. oryzae* Guy11. The plasmid pPgpdA·eGFP·hyg was used as a control. Hygromyin resistant transformants were selected for each construct and grown on complete medium plates and examined for fluorescence. For pPgpdA·eGFP·hyg, 18/20 transformants were fluorescent while only 5/100 of the pPgpdA·ACE1eGFP·hyg transformants were fluorescent.

The five *M. oryzae* transformants expressing ACE1eGFP were then grown in complete liquid medium for 7 days then extracted and examined by LCMS as described in the ESI.† All five transformants produced a new compound which was not produced by the controls. This compound had a different retention time to 12,13 dihydroxymagnaporthepyrone **6** produced in *A. oryzae* (RT = 31.6 min, Fig. 3B) and a different UV spectrum (uv_max_ = 210 nm, 248 nm, 340 nm) although the low resolution ESI MS spectrum was identical to **6**, as was the molecular formula predicted from the HRMS. Thus it appeared that this compound was isomeric to 12,13-dihydroxymagnaporthepyrone **6**. ^1^H NMR showed it to consist of a single stereoisomer, but compared to **6** it had only three contiguous olefinic CH resonances, one of which was coupled to a CHO methine which was in turn coupled to another CHO methine. The methyl doublet of **6** was replaced by a methyl doublet coupling to an olefinic CH. These data, supported by ^13^C and 2D spectra showed this compound to be 10,11-dihydroxymagnaporthepyrone **7**.

### Co-expression with the RAP1 *trans*-ER protein

Bioinformatic analysis of the sequence of the ACE1 enoyl reductase (ER) domain suggests that it is inactive, similar to the tenellin and lovastatin nonaketide synthase (LNKS) ERs – such domains are designated ER^0^ to indicate their non-catalytic role. Many fungal hrPKS which contain an ER^0^ domain recruit a *trans*-acting ER which has a catalytic and programming role. For example in the cases of pretenellin A **1**, preaspyridone **3** and lovastatin **5** biosynthesis *trans*-acting ERs encoded by *tenC*, *apdC* and *lovC* respectively interact with the PKS to provide programmed enoyl reduction and have the effect of reinforcing PKS programming including methylation and chain-length fidelity.^15^ RAP1 also encodes a *trans*-acting ER (*e.g.* RAP1 has a 48% similarity to TENC and 58% similarity to LOVC) and thus it may play a similar role in concert with ACE1, possibly controlling fidelity as well as enoyl reduction. We therefore constructed plasmids to coexpress *RAP1* with *ACE1* in both *A. oryzae* and *M. oryzae*.

For *A. oryzae* we created a plasmid carrying both the *ACE1* and *RAP1* genes in which *ACE1* lacking all introns was expressed under the control of *P*_*amyB*_, and *RAP1* was expressed from the strong constitutive alcohol dehydrogenase promoter *P*_*adh*_ from *A. oryzae* (pPamyB·ACE1[Δintrons 1–3]·argB·Padh·RAP1, Fig. 1A). *A. oryzae* M-2-3 protoplasts were transformed with pPamyB·ACE1[Δintrons 1–3]·argB·Padh·RAP1 and transformants were selected on minimal medium.

**Fig. 5:**
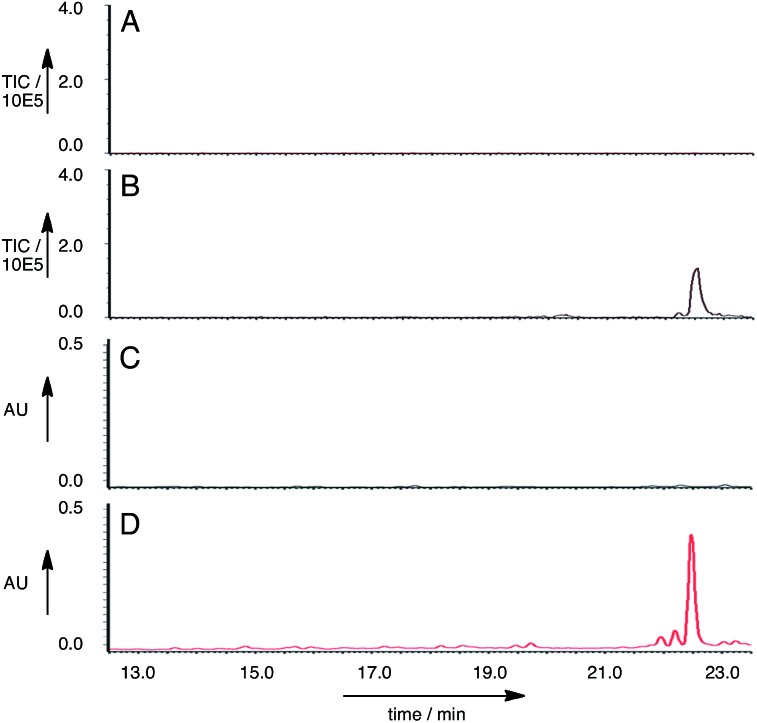
LCMS analysis of *ACE1* + *RAP1* expression: A, EIC (ES^–^) *m*/*z* 480.4 for *A. oryzae* M-2-3; B, EIC (ES^–^) *m*/*z* 480.4 for *A. oryzae* M-2-3 + *ACE1* + *RAP1*; C, uv chromatogram (280 nm) for *A. oryzae* M-2-3; D, uv chromatogram (280 nm) for *A. oryzae* M-2-3 + *ACE1* + *RAP1*.

Twenty seven of the resulting *A. oryzae* transformants were selected and transferred to new agar plates. Ten of these transformants were grown in liquid medium containing starch for seven days, then extracted in the usual way. LCMS analysis of the extracts showed that of the *A. oryzae* transformants, five produced a new peak compared to the controls in the neutral organic extract, and in lesser amounts, in the acidic and mycelial extracts. The new compound had different retention times to compounds **6** and **7** (RT = 22.5 min, uv max = 270, 280 nm, *m*/*z* 481.4 [M – H]^–^ (Fig. 5)).

**Fig. 6:**
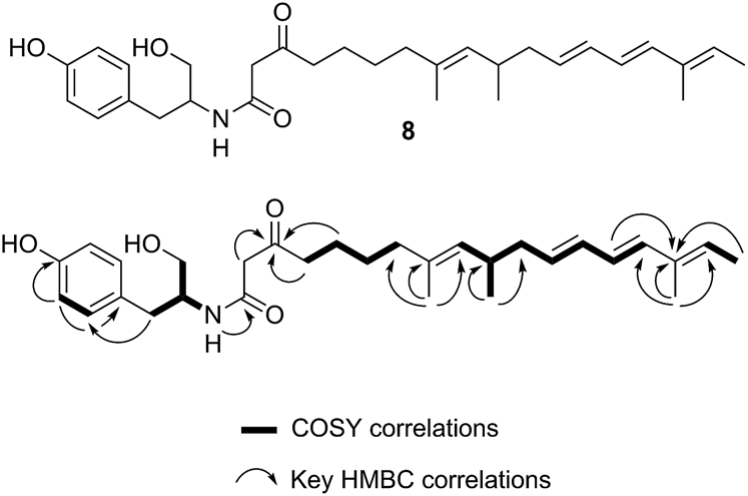
Structure and NMR data of **8** from *ACE1* + *RAP1* expression transformants.

A single *A. oryzae* transformant was grown at large scale (800 mL) to yield 65.5 mg of crude extract. The new compound was purified by HPLC and analysed by NMR and HRMS (*m*/*z* 482.3265 Da [M]H^+^, consistent with a molecular formula of C_30_H_43_NO_4_). ^1^H NMR analysis showed that the compound contained four methyl groups, six olefinic protons and an aromatic group indicative of tyrosine. ^13^C NMR, COSY, HMBC and HSQC determined the structure as **8** (Fig. 6, ESI†).

### Biological testing

**Fig. 7:**
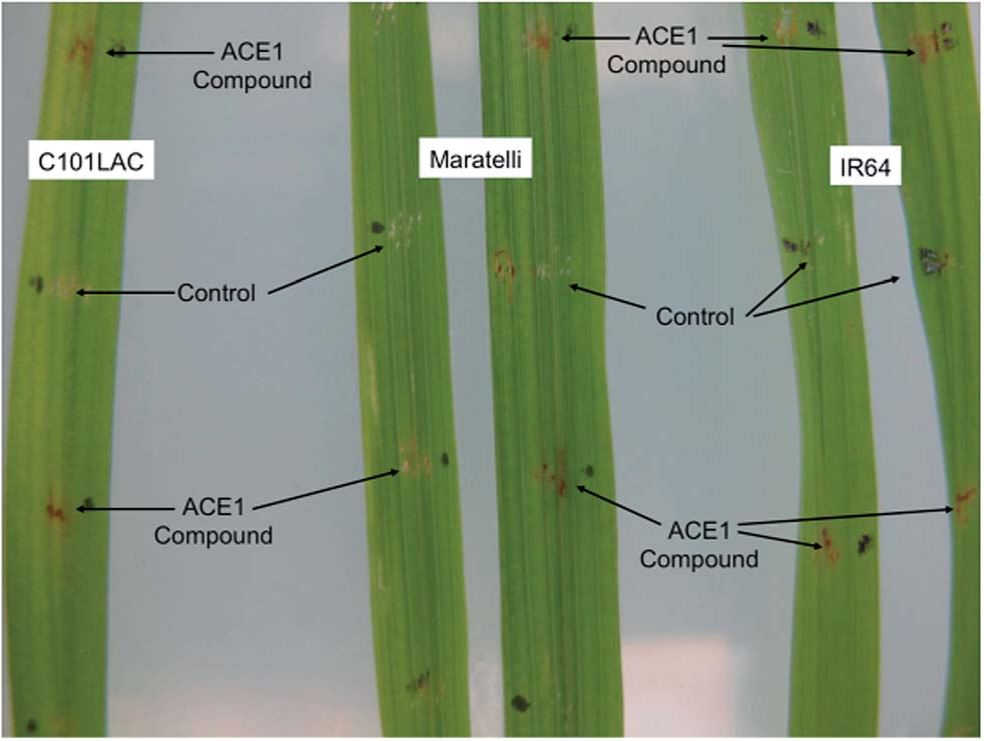
Biological testing of 12,13-dihydroxymagnaporthepyrone **6** on both resistant (IR64 and C101LAC) and susceptible (Maratelli) rice cultivars. Testing of **7** gave similar results. **8** Had no effect.

Purified magnaporthepyrones **6** and **7** were tested for biological activity on susceptible, (Maratelli, Sariceltik CO39) and resistant rice cultivars carrying *Pi33* (IR64 and C101LAC).^21^ Compounds were deposited onto normal or wounded rice leaves. Both compounds induced localised brown necrosis on all cultivars, irrespective of host genotype (Fig. 7). These results suggest that magnaporthepyrones do not induce a phenotype specifically on rice cultivars carrying *Pi33*. Addition of these compounds to spore suspensions of an isolate virulent on *Pi33* rice cultivars did not induce an AVR reaction, but slightly reduced pathogenicity on both susceptible and resistant cultivars. Compound **8** had no biological activity: no induction of leaf symptoms, no changes of a virulent interaction into AVR, no enhancement nor reduction in pathogenicity. Therefore compounds, **6**, **7** and **8** do not appear to be the avirulence signal compound produced by ACE1/*M. oryzae* Guy11 during appressorium-mediated penetration.

## Discussion

Initial attempts to express *ACE1* in *A. oryzae* produced no observable new metabolites. This was surprising as all of our previous efforts to express foreign fungal genes in this organism had been successful.

Further investigation using RT-PCR and subsequent sequence analysis clearly showed that *ACE1* intron-2 is not spliced correctly in *A. oryzae*, resulting in an mRNA with a frame-shift and premature stop codon. Intron-2 is located centrally in the PKS portion of *ACE1*, upstream of the sequence encoding the key acyl carrier protein (ACP) domain. Any truncated ACE1 protein resulting from the translation of this incorrectly spliced mRNA would not be capable of producing a polyketide product.

Removal of the *ACE1* introns resulted in transcripts which were translated correctly in *A. oryzae*. Indeed more than 75% of transformants produced full-length ACE1-eGFP protein fusions as evidenced by strong fluorescence of transformed hyphae, and the production of novel metabolites. Expression of both full-length and NRPS-deleted *ACE1* intronless constructs resulted in the production of the same compound, 12,13-dihydroxymagnaporthepyrone **6** which is exclusively of polyketide origin as shown by isotopic labelling. Expression of the same clones in *M. oryzae* resulted in the production of the closely related 10,11-dihydroxymagnaporthepyrone **7**. Similar compounds were observed by Vederas and coworkers when the lovasatin nonaketide synthase (LNKS) was expressed without its cognate ER encoded by *lovC*.^15^

**Scheme 2:**
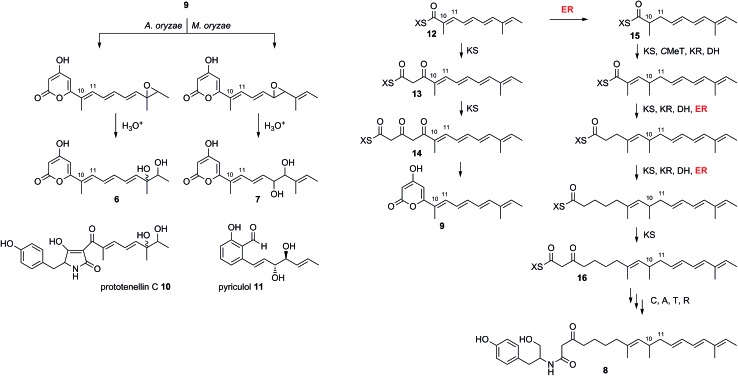
Deduced biosynthesis of ACE1 products in the presence and absence of RAP1. X = ACE1 ACP domain.

These results can be interpreted if the PKS portion of ACE1 produces the polyunsaturated pyrone **9** (which we name magnaporthepyrone, Scheme 2). This compound is then epoxidised by different monooxygenases in either *A. oryzae* or *M. oryzae* and spontaneous hydrolysis of the epoxides results in the observed diols **6** and **7**. Support for this hypothesis comes from the expression of *tenS* in *A. oryzae* where we observed the presence of prototenellin C **10** (ref. 22) which displays exactly the same chemical motif as 12,13-dihydroxymagnaporthepyrone **6** – thus *A. oryzae* must possess a monooxidase selective for the terminal methylbutenyl motif of such precursors. Likewise, *M. oryzae* is known to produce diol compounds such as pyriculol **11** (ref. 23) which must also derive from epoxidation of a polyunsaturated polyketide followed by hydrolysis. Therefore, *M. oryzae* must have a monooxygenase with selectivity for epoxidation of mid-chain polyenes.

The biosynthesis of **6** and **7** do not require an enoyl reductase step, consistent with the observation that the ACE1 ER domain is predicted to be inactive. Coexpression of *ACE1* with *RAP1*, which encodes a *trans*-acting ER, changed the biosynthetic pathway. In the heterologous host *A. oryzae*, where we constructed a single vector to carry both genes, the amide **8** was produced and compounds derived from **9** were no longer observed.

Amide **8** is closely related to pyrone **9** and its biosynthesis can be rationalised by the intervention of the RAP1 ER at the pentaketide stage of biosynthesis (Scheme 2). Pentaketide intermediate **12** is recognised by the ACE1 KS domain and extended to **13**, but in the absence of prior enoyl reduction the next required *C*MeT step is prevented. Continued chain extension by an unselective KS then gives the tricarbonyl **14** which can spontaneously offload itself from the synthase as the pyrone **9**. Alternatively, in the presence of the RAP1 ER, pentaketide **12** is reduced giving a substrate which can be extended by the KS and then correctly methylated to give **15** before following three more extension and processing cycles to give the fully extended nonaketide **16**. Thus the ACE1 *C*MeT domain appears to display an additional level of substrate selectivity in blocking correct chain processing in the absence of 10,11 reduction. Crucially, however, the KS is unselective and continues to extend the chain to the tricarbonyl **14** which can be off-loaded as the pyrone **9**. Thus the lack of selectivity by the KS effectively removes incorrectly processed chains from the PKS, preventing its blockage.

In many other investigated PKS-NRPS systems the NRPS then attaches the amino acid to the fully extended polyketide and either reductively cleaves it,^24^ or performs a non-reductive Dieckmann cyclisation reaction to release an acyl tetramic acid.^9^ In this case it appears that tyrosine is used as the amino acid and attached to the polyketide in the same way as observed in the cases of pretenellin A **1** and preaspyridone **3**. After amide formation, however, the fully elaborated product is probably reductively released. This could be *via* 2 electron reduction to form an aldehyde (as in the classic case of fungal lysine biosynthesis^25^) which could be further reduced to a primary alcohol by an adventitious *A. oryzae* enzyme, or *via* double (*i.e.* 4 electron) reduction catalysed by the NRPS R-domain itself as observed during the biosynthesis of myxochelin.^26^

Consistent with this hypothesis is the observation that the ACE1 terminal-domain has an intact conserved NADPH binding motif (GXSXXG) and the catalytic triad Ser-Tyr-Lys more closely resembles ‘*reducing*’ R-domains than DKC cyclising domains (see ESI†).^24^ Qiao *et al.* predicted a reductive release for *ccsA*, the PKS-NRPS gene involved in cytochalasin K **4** biosynthesis in *Aspergillus clavatus*,^27^ based on the same rationale, and the co-expression of *ccsA* with *ccsC* by Oikawa and co-workers led to the production of the amide **17**,^14^ also at the alcohol oxidation state.

**Scheme 3:**
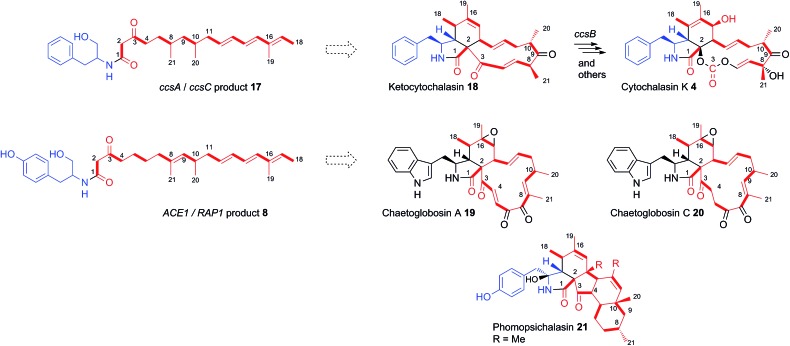
Structural relationships between isolated chemical products of fungal PKS/NRPS and *trans*-ER proteins and known natural products and biosynthetic pathways. Although cytochalasans are usually grouped according to the specific incorporated amino acid, the nature of the polyketide chain (shown in red) also contributes to their chemical diversity. Numbering for illustrative purposes only. Dashed arrow indicates biosynthetic relationship and does not indicate a direct precursor/product relationship.

The Oikawa compound **17** differs from **8** in being an octaketide rather than a nonaketide and being evidently constructed from phenylalanine rather than tyrosine (Scheme 3). A further difference is that **8** is unsaturated between carbons 8 and 9 of the polyketide, whereas **17** is fully saturated at the corresponding position. Genetic experiments have already shown that *ccsA* and *ccsC* are involved in cytochalasin K **4** biosynthesis, most likely *via* ketocytochalasin **18**.^27^ Although **17** is unlikely to be a direct precursor of **4**, it is probably a shunt metabolite from the corresponding aldehyde. Vederas, Tang and coworkers have also recently elucidated that *ccsB* from the cytochalasin K biosynthetic pathway is an FAD-dependent oxygenase which catalyses (among other reactions) the creation of the distinctive carbonate moiety of **4**.^28^

The structural similarities between the *ccsA*/*C* product **17** and the *ACE1*/*RAP1* product **8** strongly suggest that **8** is also a shunt metabolite of a cytochalasan-like biosynthetic pathway.^13^ This is supported by the observation that **8** does not show the expected biological activity as an avirulence signalling compound.

To our knowledge, only two other gene clusters have been correlated with cytochalasan biosynthesis in fungi. These are the gene clusters for chaetoglobosin A **19** biosynthesis in *Penicillium expansum* and *Chaetomium globosum*.^29,30^ Chaetoglobosin A **19** is biosynthesised from tryptophan and a nonaketide likely to be almost identical to that of amide **8**, except for the unsaturation between carbons 4 and 5. However, several chaetoglobosins are also known which are fully saturated at this position (*e.g*. chaetoglobosin C **20**)^31^ indicating that amide **8** could include the correct polyketide precursor. Analysis of the tailoring genes in the *ACE1* cluster show that they have more similarity to the chaetoglobosin biosynthetic genes than those in the *ccs* cytochalasin gene cluster (Table S2†).^32^

The nonaketide nature of the amide **8** and the fact that tyrosine is incorporated, distinguishes the *RAP1*/*ACE1* product from the *ccsA*/*ccsC* compound and mean that the likely ultimate product of the ACE1 pathway will be a member of the tyrosine-derived cytochalasans, an example of which is phomopsichalasin (also known as diaporthichalasin) **21**.^33,34^ In addition, the differences between the *ACE1* and *ccs* gene clusters, such as the lack of a *ccsB* homolog, plus additional oxidoreductases similar to those found in the chaetoglobosin A **19** gene cluster, indicate that the tailoring of the polyketide backbone is likely to be more similar to **19**, which could arise from a polyketide identical or very similar to that of **8**.

Thus we have shown that heterologous expression is an effective tool to probe cryptic gene clusters in fungi, which enables important metabolic relationships to be unravelled. It is now clear that avirulence signalling between the rice blast fungus *Magnaporthe oryzae* and rice is mediated by a natural product bearing structural similarities to the cytochalasans. Current and future work in our group is focused on exploring the enzymatic activities of the proteins encoded by other genes from the *ACE1* biosynthetic cluster to identify the bioactive compound responsible for avirulence signalling.

## Experimental

For construction of vectors, medium conditions, transformation protocols, LCMS methods, NMR data, isotope feeding experiments and details on biological testing see the ESI.†

### Characterisation of new natural products

#### 12,13-Dihydroxymagnaporthepyrone **6**

Isolated as an amorphous yellow solid (15 mg) and a 1 : 1 mixture of two diastereoisomers (A and B) by preparative HPLC. ^1^H NMR (500 MHz acetone-*d*_6_): *δ* 1.09 ppm (1.5H, d, ^3^ *J*_HH_ 6.7, H-14A), 1.11 ppm (1.5H, d, ^3^ *J*_HH_ 6.3, H-14B), 1.255 (1.5H, s, H-15A), 1.260 (1.5H, s, H-15B), 2.02 (1.5H, s, H-16A), 2.03 (1.5H, s, H-16B), 3.60 (0.5H, q, ^3^ *J*_HH_ 6.7, H-13A), 3.61 (0.5H, q, ^3^ *J*_HH_ 6.3, H-13B), 5.39 (1H, d, ^3^ *J*_HH_ 2.0, H-2A + H-2B), 6.07 (0.5H, d, ^3^ *J*_HH_ 15.3, H-11A), 6.08 (0.5H, d, ^3^ *J*_HH_ 15.3, H-11B), 6.20 (1H, d, ^3^ *J*_HH_ 2.0, H-4A + H-4B), 6.55 (1H, m, H-10AB), 6.69 (2H, m, H-8AB + H-9AB), 7.09 (1H, d, ^3^ *J*_HH_ 9.6, H-7AB); ^13^C NMR (151 MHz acetone-*d*_6_): *δ* 12.7 ppm (C-15), 18.1 & 18.3 (C-14), 24.5 & 23.7 (C-16), 74.2 (C-13), 75.8 (C-12), 90.1 (C-2), 99.0 (C-4), 126.8 (C-6), 128.2 (C-8), 129.5 & 129.8 (C-10), 132.5 (C-7), 139.6 & 139.6 (C-9), 142.8 & 143.2 (C-11), 162.4 (C-3/5), 162.6 (C-5), 163.6 (C-1), 170.7 (C-3); UV/vis *λ*_max_ (H_2_O/MeOH) 273, 362 nm; IR ν_max_ (neat) 3375 brs, 2926, 1685 br s, 1530 cm^–1^; MS (ESI^+^) *m*/*z* (%): 293 (100) [M]H^+^, 315 (50) [M]Na^+^; MS (ESI^–^) *m*/*z* (%): 291 (100) [M – H]^–^; HRMS (ESI^+^) calculated [M – H_2_O]H^+^ 275.1277 found 275.0922; HRMS (ESI^–^) calculated [M – H]^–^ 291.1033 found 291.1238.

#### 10,11-Dihydroxymagnaporthepyrone **7**

Isolated 8 mg by preparative HPLC. ^1^H NMR (500 MHz acetone-*d*_6_): *δ* 1.60 (3H, d, ^3^ *J*_HH_ 7.2, H-14), 1.65 (3H, s, H-16), 2.00 (3H, s, H-15), 3.93 (1H, d, ^3^ *J*_HH_ 7.2, H-11), 4.25 (1H, dd, ^3^ *J*_HH_ 7.7, ^3^ *J*_HH_ 7.7, H-10), 5.36 (1H, d, ^4^ *J*_HH_ 2.0,, H-2), 5.50 (1H, q, ^3^ *J*_HH_ 7.7, H-13), 6.16 (1H, d, ^4^ *J*_HH_ 2.0, H-4), 6.30 (1H, dd, ^3^ *J*_HH_ 15.7, ^3^ *J*_HH_ 15.7, H-9), 6.76 (1H, dd, ^3^ *J*_HH_ 15.2, ^3^ *J*_HH_ 15.4, H-8), 7.02 (1H, d, ^3^ *J*_HH_ 15, H-7); ^13^C NMR (151 MHz acetone-*d*_6_): *δ* 12.1 ppm (C-14), 12.8 (C-16), 13.2 (C-15), 73.7 (C-10), 81.0 (C-11), 90.8 (C-2), 99.0 (C-4), 122.1 (C-13), 126.3 (C-6), 126.3 (C-8), 132.2 (C-7), 136.8 (C-12), 142.0 (C-9), 162.0 (C-5), 163.6 (C-1), 170.8 (C-3); UV/vis *λ*_max_ (H_2_O/MeOH) 248, 340 nm; MS (ESI^+^) *m*/*z* (%): 293 (100) [M]H^+^, 315 (50) [M]Na^+^; MS (ESI^–^) *m*/*z* (%): 291 (100) [M – H]^–^; HRMS (ESI^+^) calculated [M – H_2_O]H^+^ 275.1277 found 275.0922; HRMS (ESI^–^) calculated [M – H]^–^ 291.1033 found 291.1238.

#### Amide **8**

^1^H NMR (500 MHz, CDCl_3_): *δ* 0.92 ppm (d, 3H, *J* = 6.8), 1.35 (m, 2H), 1.51 (m, 2H), 1.56 (s, 3H), 1.72 (d, 3H, *J* = 9.1), 1.74 (s, 3H), 1.95, (t, 2H, *J* = 7.5), 2.03 (t, 2H, *J* = 7.1), 2.41 (m, 1H), 2.47 (m, 2H), 2.76 (dd, 1H, *J* = 7.4, 13.9), 2.81 (dd, 1H, *J* = 7.1, 13.9), 3.31 (d, 1H, *J* = 17.2), 3.34 (d, 1H, *J* = 17.2), 3.57 (m, 1H), 3.68 (m, 1H), 4.15 (m, 1H), 4.91 (d, 1H, *J* = 9.3), 5.52 (q, 1H, *J* = 6.8), 5.61 (ddd, 1H, *J* = 14.6, 7.3, 7.3), 6.05 (dd, 1H, *J* = 14.3, 9.3), 6.08 (dd, 1H, *J* = 14.6, 9.3), 6.13 (d, 1H, *J* = 14.6), 6.75 (brd, 2H, *J* = 8),7.06 (brd, 2H, *J* = 8), 7.2 (brd, 1H, *J* = 7.8); ^13^C NMR (125 MHz, CDCl_3_): *δ* 14.6 ppm, 16.6, 18.8, 23.4, 25.4, 29.8, 35.4, 38.8, 41.9, 43.6, 46.4, 51.2, 56.1, 67, 118.1, 128.9, 129.2, 132.0, 133, 133.7, 134.6, 134.8, 135.8, 137.4, 138.1, 157.2, 169, 209.7. See ESI† for full assignments. HRMS ESI [M]H^+^: found 482.32649; predicted for C_30_H_4_3NO_4_ 482.32703.
