# Supplementary material for: Heterologous expression of the avirulence gene ACE1 from the fungal rice pathogen Magnaporthe oryzae
Source: Chem Sci. 2015 Jun 1;6(8):4837–45. doi: 10.1039/c4sc03707c (PMC5667575; doi:10.1039/c4sc03707c)
Supplement: Supplementary file 1 [file SC-006-C4SC03707C-s001.pdf]

Electronic Supplementary Material (ESI) for Chemical Science.
This journal is © The Royal Society of Chemistry 2015

Heterologous Expression of the Avirulence gene *ACE1* from the fungal rice pathogen *Magnaporthe oryzae*.

Zhongshu Song,[1a] Walid Bakeer,[1,2a] James W. Marshall,[1] Ahmed A. Yakasai,[1] Rozida Mohd Khalid,[1] Jerome Collemare,[3] Elizabeth Skellam,[4] Didier Tharreau,[5] Marc-Henri Lebrun,[6,7] Colin M. Lazarus,[8] Andrew M. Bailey,[8] Thomas J. Simpson[1] and Russell J. Cox[1,4]*

1. School of Chemistry, University of Bristol, Cantock's Close, Bristol, BS8 1TS, UK.
2. Microbiology Department, Faculty of Pharmacy, Beni Suef University, Egypt
3. UMR1345, IRHS-INRA, 49071 Beaucouzé Cedex, France;
4. Institute for Organic Chemistry, Leibniz University of Hannover, Schneiderberg 1B, 30167, Hannover, Germany
5. UMR BGPI, CIRAD, Campus International de Baillarguet 34398, Montpellier Cedex 5, France
6. UR 1290 BIOGER-CPP, INRA, Campus AgroParisTech, 78850, Thiverval-Grignon, France
7. UMR 5240 MAP, CNRS, UCB, INSA, Bayer CropScience, 69263 Lyon Cedex 09, France
8. School of Biological Sciences, University of Bristol, 24 Tyndall Avenue, Bristol BS8 1TQ, UK

1. **Construction of Vectors.**
2. **Transformation of *A. oryzae* and *M. oryzae* and fermentation procedures.**
3. **LCMS Procedures.**
4. **Characterisation of new natural products.**
5. **Biological Testing.**
6. **Reverse-Transcription Analysis of *ACE1* Introns**
7. **Bioinformatic Analysis and Discussion of the *ACE1* C-terminal domain.**
8. **Comparative analysis of the ACE1, CHGG1239 and *ccs* PKS-NRPS gene clusters**
9. **References**

1. Construction of Vectors.

Oligonucleotides synthesized and used as PCR or RT-PCR primers were purchased from Sigma-Genosys. Restriction and other pertinent sequences are marked in bold.

**Primer**	**Sequence (5' 3')**	**Purpose**
**P1**	**CACC**ATGGTGAGCAAGGGCG	*egfp* forward
**P1c**	TTACTTGTACAGCTCGTCCATGC	*egfp* reverse
**P2**	**CACC**ATGCGTGACGAGATGTGG	*ACE1* 5'
**P2c**	**AAGCTT**GCTCTCGGCAATATAC	F1 reverse primer
**P3**	**CACC**ATGCGTGACGAGATGTGGA	*ACE1* 5'
**P3c**	GCATTGAGCGAGTGGACTGGGCATTGTAATGCCTTTAGTCCGACCATCCT	intron1 spanning
**P4**	GCATTACAATGCCCAGTCCA	F5 forward
**P4c**	GCTTGACCGCCTCGAGCGTG	F5 reverse
**P5**	**CCATGG**TGAGATTCAGACAG	intron2 forward
**P5c**	TATCTCGTCGAGGTAGATGC	intron2 reverse
**P6**	CGGTGACTGGTCTCCGTCTG	intron3 forward
**P6c**	TGCACAAATTCAAGCAGCAG	intron3 reverse
**P7**	**CACC**TCCACGCCTCAACTCAACCG	F11/F12 forward
**P8**	**GTCGCTCAACGACTCGAGGAAGCTCATACGG**ATGGTGAGCAAGGGCGAGG	*egfp* overlap forward
**P9**	TAGGTCAGGGTGGTCACGAG	sequencing
**P10**	TGCGACTCACCCTGCTCAGC	T10 forward
**P10c**	AGAATGTCGAGCACTTCCTC	T10 reverse
**P11**	TGACGACAAGCCGGATATCACGTGG	T12 forward
**P12**	TTCTTCC**GCGGCCGC**CATGTACGGTCAAGATATTTTCCAA	intron2 forward
**P12c**	TTCTTCCA**CCATGG**TGCTGCACGGTCGGTCACGTCG	intron2 reverse
**P13**	TTCTTCC**GCGGCCGC**CATGATCAGGATGGTCGGACTAAAG	intron1 forward
**P13c**	TTCTTCCA**CCATGG**AGTGGACTGGGCATTGTAATGC	intron1 reverse
**P14**	TTCTTCC**GCGGCCGC**CATGAAGTGCTCGACATTCTCAAAG	intron3 forward
**P14c**	TTCTTCCA**CCATGG**ACCAGCTTTGCCGTGAATGCGT	intron3 reverese
**P15**	TTCTTC**ATGCAT**AATTCTCTTATAACCGATGATCTG	*PACE1* forward
**P15c**	TTCTTA**GCGGCCGC**GGTTGGTGATTTATTTGTTTGG	*PACE1* reverse
**P16c**	TGTACAAGAAAGCTGGGTCGGCGCGCCTTAGGTCCCGTCCTTCAGCAGTC	T12 reverse
**P17c**	TGCACAAATTCAAGCAGCAG	Recomb. site seq. primer
**P18**	GGCCTTTCCCAGACTGCTGAAGGACGGGACTAAAAGGGTGGGCGCGCCGACC	forward adaptor to remove egfp
**P19c**	TTACGCCCCGCCCTGCCACTC	reverse primer to remove egfp
**P20**	TCTTTCAACACAAGATCCCAAAGTCAAAGGATGGCGCCGTTCATTCCCTCG	forward to amplify RAP1
**P21c**	GGTTGGCTGGTAGACGTCATATAATCATACCTACAGCCGCACAACAATCTTTT	reverse to amplify RAP1

Table S1: Oligonucleotides used in this study

pENTR-D-TOPO*egfp*. The egfp gene was amplified by PCR using primers **P1** and **P1c** and pPACE1ACE1egfphyg (obtained from Dr Jerome Collemare) as the template. The product was cloned into pENTR-D-TOPO (Invitrogen) using standard procedures.

pPamyBACE1egfpargB. The plasmid pPACE1ACE1egfphyg (obtained from Dr Jerome Collemare) was digested with *Hin*dIII and *Fse*I and gel purified to give the fragment **F1**. pPACE1ACE1egfphyg was used as the template in a PCR reaction using primers **P2** and **P2c** to form a 651 bp fragment (**F2**) consisting of the 5' end of *ACE1*. **F2** was cloned into pENTR-D-TOPO and the resulting plasmid was then digested with *Fse*I and *Hin*dIII to form fragment **F3**. Fragment **F1** was ligated into **F3** to give pENTRACE1eGFP which was amplified in *E. coli*. *In vitro* Gateway LR recombination was used to mobilise *ACE1* into pTAex3GS to form pPamyBACE1egfpargB.

pEYA1ACE1[Δintron1]. The plasmid pPACE1ACE1egfphyg (obtained from Dr Jerome Collemare) was used as a PCR template to generate a fragment in which intron-1 had been removed. A PCR fragment was amplified using primers **P3** and **P3c**. The first part of **P3c** is the sequence after intron 1 that will be used as a tailed overlap region with the next fragment while the second part of the sequence is a primer binding sequence just before intron 1. The PCR product from **P3** and **P3c** was then cloned into pENTR-D-TOPO which was then used as a PCR template for amplifying fragment **F4** using primers **M13F** and **P3c**. Fragment **F5** was produced from a PCR using **P4** and **P4c**. The PCR product was cloned using Zero-blunt PCR cloning Kit and the fragment **F5** was released by *Eco*RI restriction digestion. The **F5** fragment thus contains the region starting from just after intron 1 and ended at 50 bp after the *Sma*I restriction site that will form overlap with fragment **F7**. Fragment **F7** was obtained by a *Sma*I/*Hin*dIII digest of pPACE1ACE1egfphyg followed by gel purification. pENTR-D-TOPO*egfp* was used as a template for PCR using primers **P1** and **M13R** to give fragment **F8**, which contains, at its 3' end, a region of sequence of pENTR-D-TOPO. Finally, yeast vector pEYA1 was digested with *Not*I and *Asc*I and then recombined in yeast with fragments **F4**, **F5**, **F7** and **F8** to create pEYA1ACE1[Δintron1].

pPamyBACE1[Δintrons1-3]egfpargB. Fragments **F9** (470 bp) and **F10** (469 bp) lacking introns 2 and 3 respectively were obtained by PCR from two cDNA clones of *ACE1* respectively in the region of intron 2 (5395-7434) and intron 3 (6805-9732) obtained from Dr Jerome Collemare using primer pairs **P5/P5c** and **P6/P6c** respectively. Fragment **F11** was obtained by PCR from pEYA1ACE1[Δintron1]egfp using primers **P7** and **P1c**. The yeast vector pEYA1ACE1[Δintron1] was digested with *Kpn*I and *Asc*I and gel purified and then recombined in yeast with **F9**, **F10** and **F11** to create pEYA1ACE1[Δintrons1-3]egfp. This in turn was LR recombined with pTAex3·GS[1] to create pPamyBACE1[Δintrons1-3]egfpargB.

pPamyBACE1[Δintrons1-3]argB. The plasmid pEYA1ACE1[Δintrons1-3]egfp was used as a template in a PCR reaction using primers **P18** and **P19c** to give **T24**. The plasmid pEYA1ACE1[Δintrons1-3]egfp was linearised with *Asc*1 and then recombined with **T24** in yeast to give the plasmid pEYAACE1[Δintrons1-3]stop. Gateway *in vitro* recombination was then used to move *ACE1* from pEYAACE1[Δintrons1-3]stop into the destination vector pTAYA-GSpAGE/T2[1] to form pPamyBACE1[Δintrons1-3]argB.

pPamyBACE1[Δintrons 1-3]argBPadhRAP1. The *RAP1* gene was amplified from pPniaDRAP1bar using primers **P20** and **P21c** to give **T25**. The vector pTAYA-GSpAGE/T2[1] was linearised with *Asc*1 and recombined with **T25** in yeast to form the destination vector pTAYA-GS-RAP1. Gateway *in vitro* recombination was then used to move ACE1[Δintrons1-3]stop from pEYAACE1[Δintrons1-3]stop into pTAYA-GS-RAP1 to form pPamyBACE1[Δintrons 1-3]argBPadhRAP1.

pAmyBACE1pks[Δintrons1-2]egfpargB. The *egfp* gene was amplified using primers **P8** and **M13R** using pEYAACE1[Δintron1]egfp as a template and then gel purified to give **T8**. pEYA1ACE1[Δintron1]was digested with *Mlu*I and the largest piece **T2** was gel purified. **T1, T2**, and **T8** concentrations were adjusted to be equaimolar following gel electrophoresis analysis. The latter three DNA pieces were transformed in yeast for recombination. The recombined plasmid extracted from yeast was cloned in *E. coli* for amplification and then extracted from five clones. Recombined plasmids extracted were tested for correctness *via* restriction analysis using *Asc*I, *Nde*I, and *Sma*I. Correctly recombined plasmid pEYA1*ACE1E1E2I2E3I3E4(518)·egfp* was sent for sequencing the fusion site between *egfp* and the *ACE1*-part using primer **P9**. Intron-2 containing sequence was removed from pEYA1*ACE1E1E2I2E3I3E4(518)·egfp* using digestion with *Bae*I and the cut construct was gel purified to give **T9**. Intron-2 free *ACE1* PCR product (corresponding to and larger than the removed one and hence recombinable with **T9**) was amplified using primers **P10** and **P10c** and gel purified to give **T10**. Overlapping cDNA provided by Dr. Jerome Collemare, which were amplified by RT-PCR across intron-2 and intron-3 area using RNA extracted during appressorial expression of *ACE1*, were used as the template in the latter PCR. **T9** and **T10** were then recombined in yeast. The recombined plasmid extracted from yeast was cloned in *E. coli* for amplification and then extracted from five clones. Recombined plasmids extracted were tested for correctness *via* restriction analysis using *Asc*I, *Nde*I, and *Sma*I. Correctly recombined plasmid pEYA1*ACE1E1E2E3I3E4(518)egfp* was sequenced using **P10** and **P10c** primers to confirm intron-2 removal.

pEYA1*ACE1E1E2E3I3E4(518)egfp* was LR recombined with pTAex3·GS[1] leading to pAmyBACE1pks[Δintrons1-2]egfpargB.

pPamyBACE1[Δintrons1-2]argB. The vector pEYA1ACE1E1E2E3I3E4(518)egfp was digested with *Asc*I and gel purified to give **T11**. The NRPS encoding sequence of ACE1 was amplified using **P11** and **P16c** primers and pPACE1ACE1egfphyg as the template to give **T12** which was then gel purified. **T11** and **T12** were then recombined in yeast. The recombined plasmid extracted from yeast was cloned in *E. coli* for amplification and then extracted from five clones. Recombined plasmids extracted were tested for correctness *via* restriction analysis using *Asc*I, *Nde*I, and *Sma*I. The site of recombination between **T11** and **T12** on the *ACE1* sequence was checked by sequencing using primer **P17c**. The confirmed plasmid pEYA1ACE1E1E2E3I3E4 was LR recombined with pTAex3·GS[1] leading to **pPamyBACE1[Δintrons1-2]argB.**

pUC18hygPgpdAGS. The hph cassette (hygromycin resistance gene driven by the *PtrpC* promoter) was cloned into pUC18 at its *Kpn*I site to create pUC18hyg. The *PgpdA* promoter was amplified by PCR using primers modified with *Hin*dIII and *Bam*HI restriction sites at their 5' termini. The PCR product was cloned into pCR4Blunt-TOPO (Invitrogen) which was then digested with *Hin*dIII and *Bam*HI to give a 2164 bp fragment **R1**. The vector pUC18hyg was digested with *Hin*dIII and *Bam*HI and gel purified to give a 4076 bp product **R2**. **R1** was ligated into **R2** to give pUC18hygPgpdA. The plasmid pUC18hygPgpdA was then digested with *Hin*dIII and gel purified to create **R3**. A Gateway cassette (*i.e. att*R1Cm^{R}*ccdbatt*R2) was amplified by PCR using primers each with *Hin*dIII restriction sites at their 5' termini – this fragment was gel purified, digested with *Hin*dIII and then ligated into **R3** to create two vectors: pUC18hygPgpdAGS in which the Gateway cassette is cloned in the *sense* direction; and pUC18hygBPgpdAGS(anti) in which the Gateway cassette is cloned in the *antisense* direction.

pPgpdAACE1pks[Δintrons1-2]egfphyg. pEYA1*ACE1E1E2E3I3E4(518)·egfp* was LR recombined with pUC18hygBPgpdAGS to give pPgpdAACE1pks[Δintrons1-2]egfphyg.

pPgpdaACE1egfphyg. pENTRACE1eGFP was LR recombined with pUC18hygBPgpdAGS to form pPgpdaACE1egfphyg.

pPniaDRAP1bar. This was obtained from Dr Jerome Collemare.[2]

pPamyB·intron1egfp·argB. pENTR-D-TOPO*egfp* was digested with *Not*I and *Nco*I and gel purified to give **T15**. *ACE1* intron-1 with 21 bp of exon-1 at its 5'-end and with 24 bp of exon-2 following its 3'-end was amplified using **P13** and **P13c** primers and pPACE1ACE1egfphyg was used as a template. **P13** has a *Not*I site and **P13c** has an *Nco*I site, therefore, the amplified latter fragment was digested with both enzymes and then gel purified to give **T16**. **T16** was cloned into **T15** *via* ligation and then transformed into *E. coli* which was selected on LB plates with kanamycin. The recombined plasmid pENTR*E1[21]I1E2[24]·egfp* was confirmed by sequencing using **M13F** primer. pENTR*E1[21]I1E2[24]·egfp* was LR recombined with pTAex3·GS[1] leading to pPamyB·intron1egfp·argB.

pPamyB·intron2egfp·argB. *ACE1* intron-2 with 21 bp of exon-2 at its 5'-end and with 24 bp of exon-3 following its 3'-end was amplified using **P12** and **P12c** primers and pPACE1ACE1egfphyg as a template. **P12** has a *Not*I site and **P12c** has an *Nco*I site, therefore, the amplified latter fragment was digested with both enzymes and then gel purified to give **T17**. **T17** was cloned into **T15** *via* ligation and then transformed into *E. coli* which was selected on LB plates with kanamycin. The recombined plasmid pENTR*E2[21]I2E3[24]·egfp* was confirmed by sequencing using **M13F** primer. pENTR*E2[21]I2E3[24]·egfp* was LR recombined with pTAex3·GS[1] leading to pPamyB·intron2egfp·argB.

pPamyB·intron3egfp·argB. *ACE1* intron-3 with 21 bp of exon-3 at its 5'-end and with 24 bp of exon-4 following its 3'-end was amplified using **P14** and **P14c** primers and pPACE1ACE1egfphyg as a template. **P14** has a *Not*I site and **P14c** has an *Nco*I site, therefore, the amplified latter fragment was digested with both enzymes and then gel purified to give **T18**. **T18** was cloned into **T15** *via* ligation and then transformed into *E. coli* which was selected on LB plates with kanamycin. The recombined plasmid pENTR*E3[21]I3E4[24]·egfp* was confirmed by sequencing using **M13F** primer. pENTR*E3[21]I3E4[24]·egfp* was LR recombined with pTAex3·GS[1] leading to pPamyB·intron3egfp·argB.

pPgpdA·intron1egfp·hyg. This construct was established by LR recombination of pENTR*E1[21]I1E2[24]·egfp* with pUC18*hygBPgpdA·GS*.

pPgpdA·intron2egfp·hyg. This construct was established by LR recombination of pENTR*E2[21]I2E3[24]·egfp* with pUC18*hygBPgpdA·GS*.

pPgpdA·intron3egfp·hyg. This construct was established by LR recombination of pENTR*E3[21]I3E4[24]·egfp* with pUC18*hygBPgpdA·GS*.

pPACE1·intron1egfp·hyg. The *ACE1* promoter was amplified using **P15** and **P15c** with p*PACE1egfp*hyg used as a template. **P15** has an *Nsi*I site and **P15c** has a *Not*I site, therefore, the amplified latter fragment was digested with both enzymes and then gel purified to give **T19**. pPgpdA·intron1egfp·hyg was then digested with *Nsi*I and *Not*I and gel purified to give **T20**. **T19** was then cloned by ligation into **T20** leading to pPACE1·intron1egfp·hyg.

pPACE1·intron2egfp·hyg. pPgpdA·intron2egfp·hyg was digested with *Nsi*I and *Not*I and gel purified to give **T21**. **T19** was then cloned by ligation with **T21** leading to pPACE1·intron2egfp·hyg.

pPACE1·intron3egfp·hyg. pPgpdA·intron3egfp·hyg was digested with *Nsi*I and *Not*I and gel purified to give **T22**. **T19** was then cloned by ligation with **T22** leading to pPACE1·intron3egfp·hyg.

pPgpdA·egfp·hyg. This construct was established by LR recombination of pENTR-D-TOPO·*egfp* with pUC18*hygBPgpdA·GS*

pPACE1·egfp·hyg. pPgpdA·egfp·hyg was digested with *Nsi*I and *Not*I to remove the PgpdA sequence and gel purified to give **T23**. **T19** was then cloned by ligation with **T23** leading to pPACE1·egfp·hyg.

2. Transformation of *A. oryzae* and *M. oryzae*

2.1 Media

DPY agar medium. 20 g dextrin, 10 g polypeptone, 5 g yeast extract, 5 g KH_2PO_4, 0.5 g $MgSO_4$, and 15 g agar were dissolved in 1 liter of distilled water and sterilized by autoclaving at 121 °C for 15 minutes at 15 psi. The DPY agar plates were inoculated with 1 to 2 mL of *A. oryzae* spores suspension. Inoculated plates were incubated for 13 to 15 days at 28 °C until sporulation which was assessed by inspection under light microscope.

Complete medium (CM). The CM broth medium was prepared by dissolving 10 g glucose, 2 g peptone, 1 g yeast extract, 1 g casamino acids, 6 g $NaNO_3$, 0.5 g KCl, 0.5 g $MgSO_4$, and 1.5 g KH_2PO_4 in 1 litre distilled water. The pH was adjusted to 6.5. This is followed by autoclaving at 121 °C for 15 minutes at 15 psi. Filter sterilized trace elements solution (7.9 $mg \cdot L^{-1}$ $ZnSO_4.7H_2O$, 0.6 $mg \cdot L^{-1}$ $CuSO_4$, 0.1 $mg \cdot L^{-1}$ H_3BO_3, 0.2 $mg \cdot L^{-1}$ $MnSO_4.H_2O$, and 0.14 $mg \cdot L^{-1}$ $NaMoO_4$) was added in a final concentration of 0.1 % (*v/v*) to the latter medium after cooling down. Additionally, filter sterilized vitamin supplement (1 $mg \cdot L^{-1}$ thiamine, 5 $mg \cdot L^{-1}$ biotin) was added in a final concentration of 0.1 % (*v/v*). CM broth was prepared in 100 mL aliquots dispensed in 500 mL Erlenmeyer conical flasks.

The autoclaved flasks containing the CM broth were inoculated with *M. oryzae* spores suspension for preparing to transformation. However, CM broth used for fermentation with purpose of compound production was modified by increasing sugar concentrations to 20 $g \cdot L^{-1}$ of glucose and 10 $g \cdot L^{-1}$ of sucrose. The inoculated flasks were incubated at 25 °C for 2 - 7 days according to the experiment design.

CM agar medium was made by inclusion of 15 $g \cdot L^{-1}$ of agar to the CM broth medium prior to autoclaving and processed in the same way as previously mentioned. The *M. oryzae* inoculated plates were incubated at 25 ˚C for 10-12 days until growth and sporulation were obvious under microscope. Mycelia and spores were then collected by adding 2-3 mL of sterile water and scrapping the plates to inoculate different media. Storage was made by mixing the latter spore suspension as 1:1 to sterilized 40 % glucerol and stored at - 80 °C.

STC buffer, 200 g·L^{-1} sucrose, 50 mM Tris-HCl, 50 mM $CaCl_2.2H_2O$, pH 7.5.

PTC buffer, 60 % PEG 3350, 1 M sorbitol, 50 mM Tris-HCl, 50 mM $CaCl_2.2H_2O$, pH 7.5.

TB3 recovery medium, 200 g·L^{-1} sucrose, 3 g·L^{-1} yeast extract.

TNK-CP agar medium. The TNK-CP agar medium was made by dissolving 10 g glucose, 2 g yeast extract, 15 g agar, 2 g $NaNO_3$, 2 g KH_2PO_4, 0.5 g $MgSO_4.7H_2O$, 0.1 g $CaCl_2.2H_2O$, and 4 mg $FeSO_4.7H_2O$ in 1 litre of distilled water. The pH of the medium is adjusted to 5.5 - 5.8. This is followed by autoclaving at 121 °C for 15 minutes at 15 psi. Filter sterilized trace elements solution and filter sterilized vitamin supplement (see CM medium) were added in concentration of 0.1 % (*v/v*) each to the latter medium after cooling down. They were added before dispensing the medium into plates.

TNK-CP-Su-Hyg medium. TNK-CP-Su-Hyg has the same components of and prepared in the same way as TNK-CP medium with the addition of 200 g·L^{-1} sucrose before autoclaving and addition of hygromycin B in a final concentration of 240 mg·L^{-1} before use.

TNK-CP-Hyg medium. TNK-CP-Hyg has the same components and is prepared in the same way as TNK-CP medium with the addition of hygromycin B in a final concentration of 120 mg·L^{-1} before use.

Glucose medium. 20 g glucose, 10 g peptone and 30 g sucrose containing CD for *A. oryzae* transformants.

Starch medium. 20 g starch and 10 g peptone containing CD for *A. oryzae* transformants and CM for *M. oryzae* transformants.

2.2 Transformation of *A. oryzae*.

A 150 µL spore suspension of *A. oryzae* strain M-2-3 was spread onto DPY agar plates incubated at 30 °C for 7 - 10 days. Sterile water (5 mL) was added and the spores scraped off with a sterile loop. The liquid was collected and inoculated into DPY growth medium (100 mL) which was then incubated at 30 °C with shaking (200 rpm, 24 h). Mycelia were collected by filtration through sterile filter paper, washed with sterile water, then washed with 0.9 M sodium chloride, and centrifuged (10 000 *g*, 10 min) and the supernatant was discarded. Filter-sterilized protoplasting solution (*Trichoderma harzianum*

lysing enzymes, 20 mg·mL^{-1}; Driselase enzyme, 10 mg·mL^{-1}; NaCl, 0.9 M, 20 mL) was added to the biomass which was resuspended thoroughly by vortexing.

The tube was incubated at 25 °C, with gentle mixing on a rotator. Sufficient protoplast formation was checked by microscope every 30 min. The protoplasts were filtered through sterile Miracloth. The filtrate was gently centrifuged (700 g, 5 min) and the supernatant was removed. The obtained protoplasts were washed with 0.9 M NaCl and with Solution I gently and centrifuged (700 g, 5 min). The concentration of protoplasts was determined by using a haemocytometer (Fisher). The protoplasts were resuspended in Solution I (0.9 M NaCl, 50 mm $CaCl_2$, 50 mm Tris-HCl, pH 7.5) to give 2.5×10^8 protoplasts·mL^{-1} and stored on ice. Then Solution II (60% PEG 3350, 0.9 M NaCl, 50 mM $CaCl_2$, 50 mM Tris-HCl, pH 7.5) (about 1/5 volume of the protoplast solution) was added and mixed gently.

Then 0.2 mL of the obtained mixture was added into 10 mL sterile tubes. DNA (5-10 µg, 20 µL maximum) to be transformed into the fungus was added to the 0.2 mL protoplast suspension and incubated on ice (30 min). Solution II (1 mL) was added and mixed gently, then incubated at room temperature (20 min). Czapek-Dox agar (Oxoid) in sorbitol (1 M; 8 mL; lower than 45 °C) was added to the transformation mixture and overlaid onto Czapek-Dox agar with sorbitol (1 M; 15 mL) plates. The plates were incubated at 30 °C for 4 - 6 days. The transformants were picked using a sterile toothpick and transferred to Czapek-Dox agar plates. Two rounds of selection on Czapek-Dox media were further performed. The genuine *argB* transformants were then grown on DPY agar for 7 - 10 days for spore production. These transformants were then stored at 4 °C and selected for chemical analysis.

2.3 Transformation of *M. oryzae*.

M. oryzae strain used in this research was the Guy11 strain known to be sensitive to both hygromycin B as well as Basta antibiotics. Constructs used for transformation and expression in *M. oryzae* Guy11 contained the *hygB* or *bar* cassettes.

M. oryzae was grown on CM plates for 10 - 12 days. A plug of the medium with fungal mycelia was aseptically transferred to 50 - 100 mL sterilized CM broth in a 500 mL Erlenmeyer conical flask. The flask was incubated at 25 ˚C for 3 - 4 days with shaking at 150 rpm. Mycelia were homogenised (IKA T18 basic homogonizer) and transferred to a new CM broth medium followed by incubation under the same conditions for 1 - 2 days. Mycelia were collected by filtration, and protoplasting was performed using *Trichoderma harzianum* lysing enzymes (20 mg·mL^{-1}) and Driselase enzyme (10 mg·mL^{-1}) in NaCl (0.9 M) with gentle shaking at room temperature for 2 - 3 h. Protoplasts were filtered through miracloth and centrifuged at 2000 rpm for 10 min. Protoplasts were then resuspended (washed) in sterilized STC buffer and centrifuged again at 2000 rpm for 10 minutes.

Protoplasts were diluted to 3 x $10^7 \cdot mL^{-1}$ in STC buffer and approximately 5 µg (20 µl) of appropriate plasmid was added to 200 mL protoplast solution. The mixture was incubated on ice for 30 min, 1 mL of filter sterilized PTC buffer was then added and the mixture was incubated at room temperature for a further 30 min. 4 – 5 mL of sterilized TB3 recovery medium was added to the concentrate and left to be shaken very gently overnight. The mixture was centrifuged at 3000 rpm and supernatant was reduced to 1 mL for concentration. The concentrate was then mixed with 19 mL of molten TNK-SU-CP-Hyg containing 240 $\mu g \cdot mL^{-1}$ hygromycin B at 45 - 50 C and mixed well then poured in plates. Plates were incubated at 25 - 28 C for 5-7 days with daily observation until most or all colonies of transformants appeared. Transformants were then further selected on TNK-CP-Hyg plates (2^{ndry} transformation plates) containing 120 $\mu g \cdot mL^{-1}$ hygromycin B. Finally, clones grown on 2^{ndry} plates were purified to single spores and used or stored.

Co-transformation of *M. oryzae* Guy11 with both *hygB* containing *ACE1* construct with pNIA-*RAP1* containing *bar* gene was achieved in two stages. An *ACE1* expressing *M. oryzae* transformant was transformed with the pNIA-*RAP1* following the method mentioned above. Basta in a concentration 35 $\mu g \cdot mL^{-1}$ was used in addition to hygromycin B for the selection of these co-transformants.

2.4 Fermentation of *A. oryzae* transformants and extraction

Sterile water (5 mL) was added to the transformant plate and the spores scraped off with a sterile loop. The suspensions (200 µL) obtained from transformants were inoculated into glucose medium (100 mL) to repress expression. The flasks were incubated at 30 °C with shaking at 200 rpm for 3 days. The medium was then replaced with fresh liquid minimal media containing starch for induction and incubated at 30 °C with shaking at 200 rpm for 3 days.

The culture broth was acidified using 2 M hydrochloric acid until the solution reached pH 4.0. The mycelia were homogenised using a hand-held kitchen blender and separated by filtration. The obtained broth was extracted three times with 1 volume of ethyl acetate. The mycelia were stirred in acetone for 12 h, filtered to remove solids. The acetone was removed by evaporation at reduced pressure and the remaining aqueous layer was extracted with ethyl acetate. The ethyl acetate extract, after being dried over anhydrous MgSO4, was evaporated at reduced pressure. The residue was dissolved in 2 mL methanol, diluted and used for LC-MS analysis.

2.5 Modified extraction of *A. oryzae* transformants

After fermentation, the resultant culture was homogenised using a hand-held kitchen blender and vacuum- filtered through filter paper. The liquid medium was extracted by equal amount of ethyl

acetate once. The medium was acidified to pH 4-5 by 2M HCl and extracted again with an equal volume of ethyl acetate. The two parts of ethyl acetate extract were combined, dried over anhydrous $MgSO_4$ and the liquid was evaporated at reduced pressure. The residue was dissolved in MeOH for LCMS analysis (referred to as "medium" samples). Additionally, the cell filtrate was placed in acetone for 2 hours whilst stirring. The acetone was evaporated at reduced pressure and the remaining liquid was extracted once with 50 mL of ethyl acetate. The resultant organic layer was dried over anhydrous $MgSO_4$ and the liquid was evaporated at reduced pressure. Afterwards, the dried compounds were dissolved in 2 mL methanol, diluted and used for LC-MS analysis (referred to as "cells" samples).

2.6 Fermentation of *M. oryzae* transformants and extraction

M. oryzae transformants with *ACE1* constructs were inoculated in a sterilized modified CM broth (100 mL 7 Erlenmeyer flasks with 500 mL capacity) for 6 - 7 days at 25 °C and 150 rpm shaking condition. *M. oryzae* co-transformants with *ACE1* and *RAP1* were inoculated under the same condition. Basta antibiotic was added to the test flasks to keep selection of transformants, while flasks inoculated with contol fungi were not supplemented with Basta. The cultures were then segregated into mycelia fraction and aqueous phase which were treated, extracted, evaporated and analysed the same way as *A. oryzae*. The pyrone from *M. oryzae* was extracted from both the aqueous phase and the separated mycelia.

3. LCMS Procedures

Samples were dissolved in MeOH to 1 mg·mL^{-1}, centrifuged for 5 min at 13, 500 rpm, and the supernatant was analysed by LCMS using a Waters 2795HT HPLC system. Detection was achieved by uv between 200 and 400 nm using a Waters 2998 diode array detector, and by simultaneous electrospray (ES) mass spectrometry using a Waters ZQ spectrometer detecting between 150 and 600 *m/z* units. Chromatography (flow rate 1 mL·min^{-1}) was achieved using either Phenomenex LUNA column (5 μ, C_{18}, 100 Å, 4.6 × 250 mm) equipped with a Phenomenex Security Guard precolumn (Luna C_5 300 Å) or a Phenomenex Kinetex column (2.6 μ, C_{18}, 100 Å, 4.6 × 100 mm) equipped with a Phenomenex Security Guard precolumn (Luna C_5 300 Å). Solvents were: **A**, HPLC grade H_2O containing 0.05% formic acid; **B**, HPLC grade MeOH containing 0.045% formic acid; and **C**, HPLC grade CH_3CN containing 0.045% formic acid). Gradients were as follows.

Method 1. Luna/MeOH: 0 min, 25% **B**; 5 min, 25% **B**; 51 min, 95% **B**; 53min, 95% **B**; 55 min, 25% **B**; 59 min, 25% **B**; 60 min, 25% **B**.

Method 2. Luna/CH_3CN: 0 min, 5% **C**; 5 min, 5% **C**; 45 min, 75% **C**; 46 min, 95% **C**; 50 min, 95% **C**; 55 min, 5% **C**; 60 min, 5% **C**.

Method 3. Kinetex/MeOH: 0 min, 10% **B**; 10 min, 90% **B**; 12 min, 90% **B**; 13min, 10% **B**; 15 min, 10% **B**.

Method 4. Kinetex/CH_3CN: 0 min, 10% **C**; 10 min, 90% **C**; 12 min, 90% **C**; 13min, 10% **C**; 15 min, 10% **C**.

Semi-Preparative LCMS and compound purification.

Purification of compounds was generally achieved using a Waters mass-directed autopurification system comprising of a Waters 2767 autosampler, Waters 2545 pump system, a Phenomenex LUNA column (5μ, C_{18}, 100 Å, 10 × 250 mm) equipped with a Phenomenex Security Guard precolumn (Luna C_5 300 Å) eluted at 4 mL/min at ambient temperature. Solvent A, HPLC grade H_2O + 0.05% formic acid; Solvent B, HPLC grade CH_3CN + 0.045% formic acid. The post-column flow was split (100:1) and the minority flow was made up with MeOH + 0.045% formic acid to 1 $mL \cdot min^{-1}$ for simultaneous analysis by diode array (Waters 2998), evaporative light scattering (Waters 2424) and ESI mass spectrometry in positive and negative modes (Waters Quatro Micro). Detected peaks were collected into glass test tubes. Combined tubes were evaporated under a flow of dry N_2 gas, weighed, and residues dissolved directly in NMR solvent for NMR analysis.

30 minute ramp program: 5% B to 60% B for magnaporthepyrones purification, 45% B to 95% B for compound 8 purification over 25 min, 95%B to 5%B over 2 min, hold for 5 min.

4. Characterisation of new natural products.

12,13-Dihydroxymagnaporthepyrone 6. Isolated as an amorphous yellow solid (15 mg) and a 1:1 mixture of two diasteroisomers (A and B) by preparative HPLC. ^{1}H NMR (500 MHz Acetone-d$_6$): δ 1.09 ppm (1.5H, d, $^{3}J_{HH}$ 6.7, H-14A), 1.11 ppm (1.5H, d, $^{3}J_{HH}$ 6.3, H-14B), 1.255 (1.5H, s, H-15A), 1.260 (1.5H, s, H-15B), 2.02 (1.5H, s, H-16A), 2.03 (1.5H, s, H-16B), 3.60 (0.5H, q, $^{3}J_{HH}$ 6.7, H-13A), 3.61 (0.5H, q, $^{3}J_{HH}$ 6.3, H-13B), 5.39 (1H, d, $^{3}J_{HH}$ 2.0, H-2A + H-2B), 6.07 (0.5H, d, $^{3}J_{HH}$ 15.3, H-11A), 6.08 (0.5H, d, $^{3}J_{HH}$ 15.3, H-11B),6.20 (1H, d, $^{3}J_{HH}$ 2.0, H-4A + H-4B), 6.55 (1H, m, H-10AB), 6.69 (2H, m, H-8AB + H-9AB), 7.09 (1H, d, $^{3}J_{HH}$ 9.6, H-7AB); ^{13}C NMR (151 MHz Acetone-d$_6$): □ 12.7 ppm (C-15), 18.1 & 18.3 (C-14), 24.5 & 23.7 (C-16), 74.2 (C-13), 75.8 (C-12), 90.1 (C-2), 99.0 (C-4), 126.8 (C-6), 128.2 (C-8), 129.5 & 129.8 (C-10), 132.5 (C-7), 139.6 & 139.6 (C-9), 142.8 & 143.2 (C-11), 162.4 (C-3/5), 162.6 (C-5), 163.6 (C-1), 170.7 (C-3); UV/vis $□_{max}$ (H_2O/MeOH) 273, 362 nm; IR $□_{max}$ (neat) 3375 brs, 2926, 1685 br s, 1530 cm^{-1}; MS (ESI$^+$) *m/z* (%): 293 (100) [M]H$^+$, 315 (50) [M]Na$^+$; MS (ESI$^-$) *m/z* (%): 291 (100) [M - H]$^-$; HRMS (ESI$^-$) calculated [M - H]$^-$ 291.1238; found 291.1238.

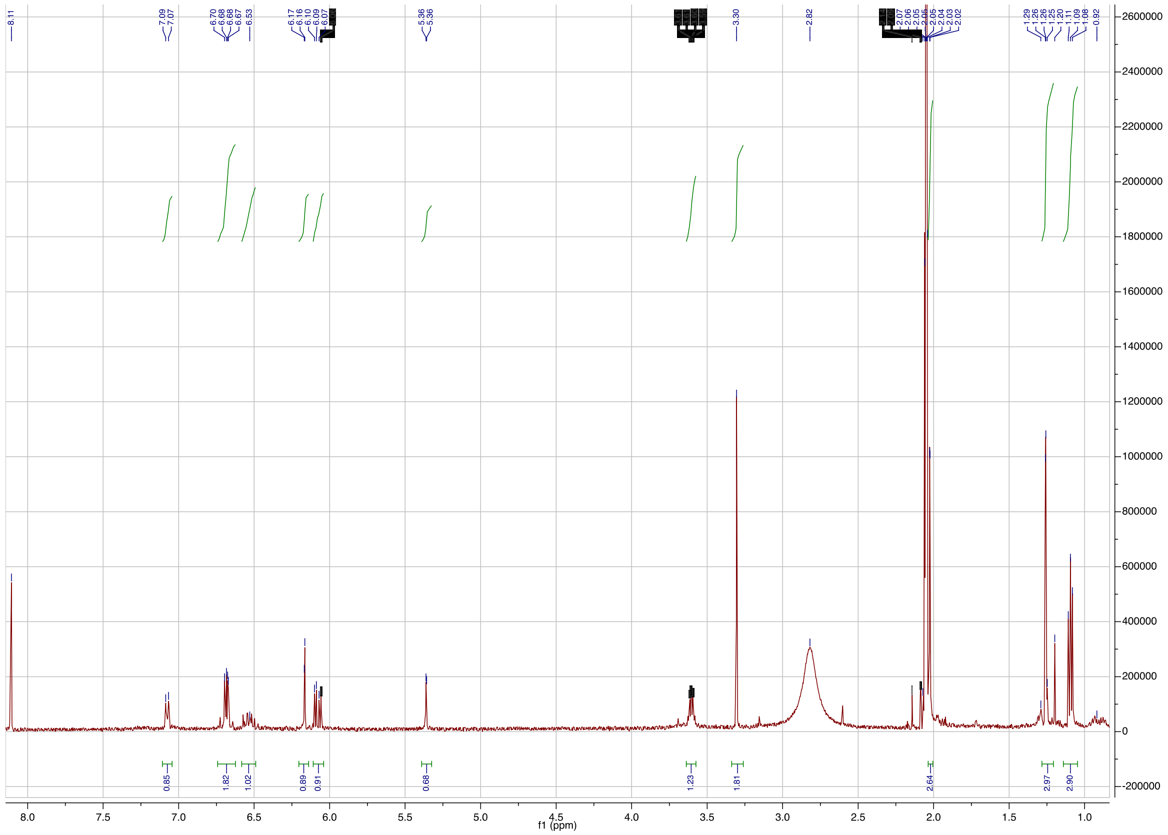

Figure S1A. ^{1}H NMR of **6**.

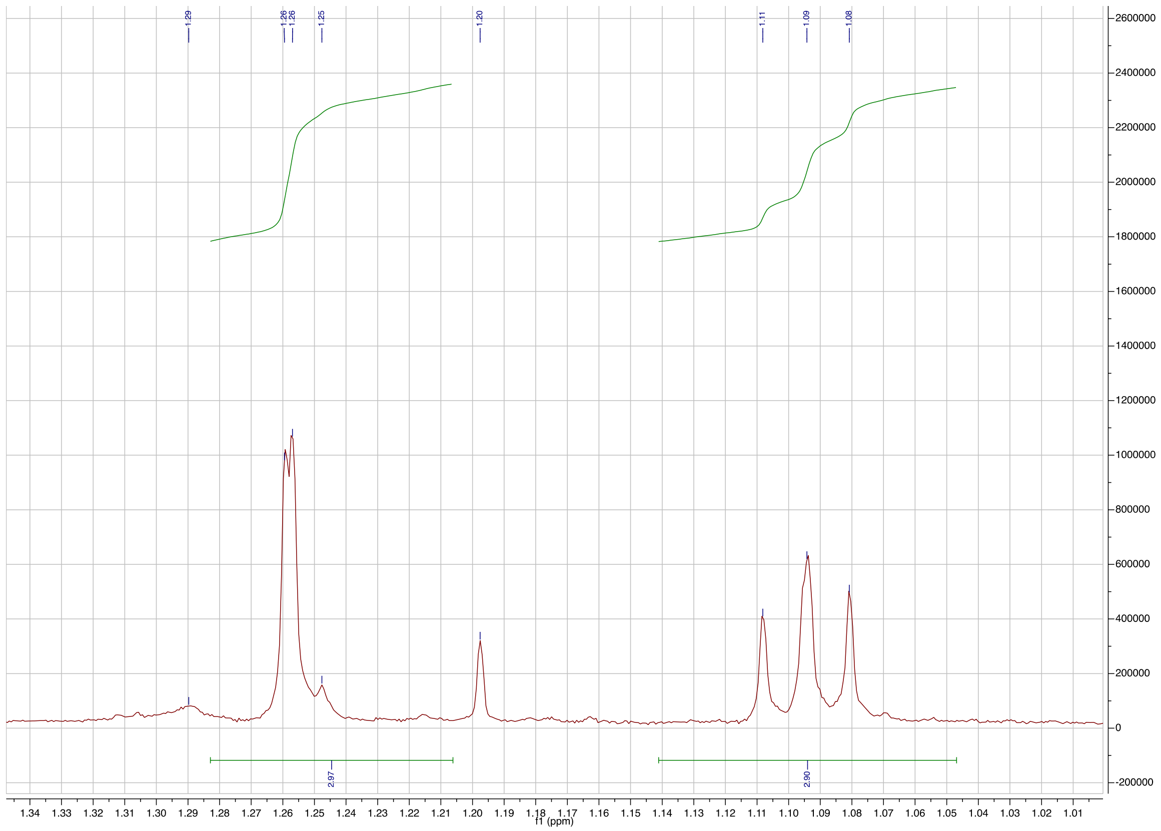

Figure S1B. ^{1}H NMR of **6**.

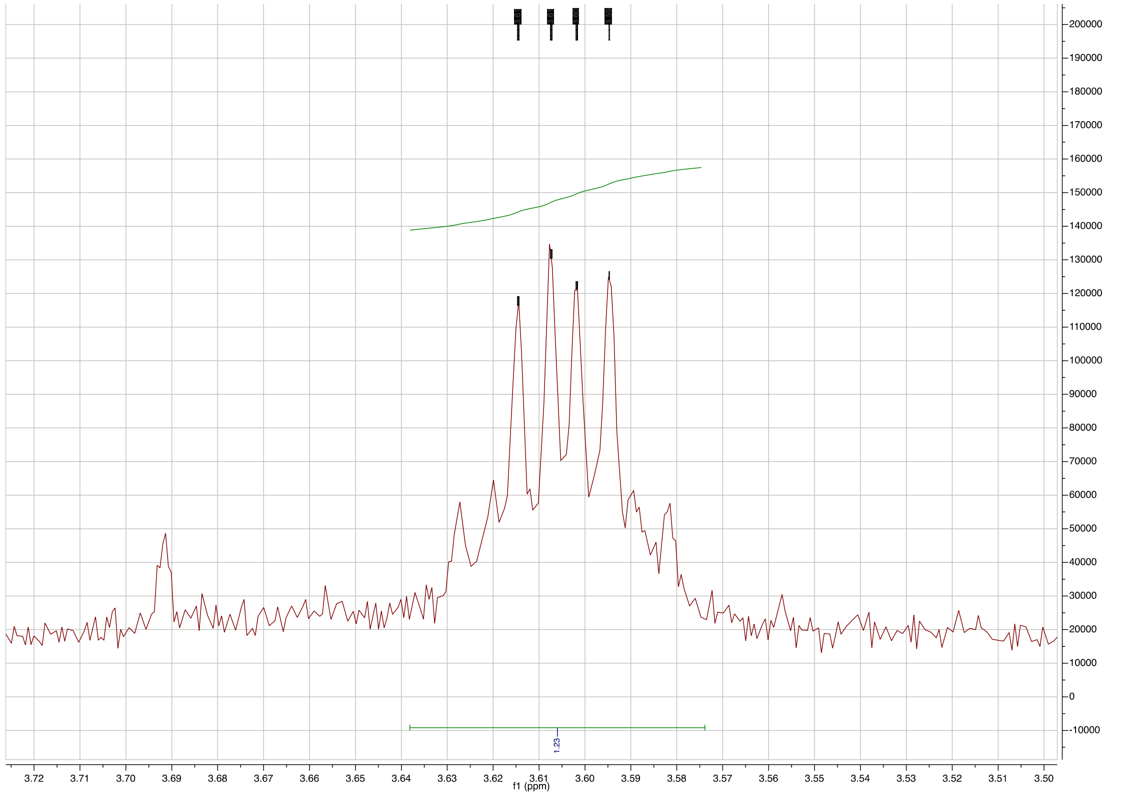

Figure S1C. ^{1}H NMR of **6**.

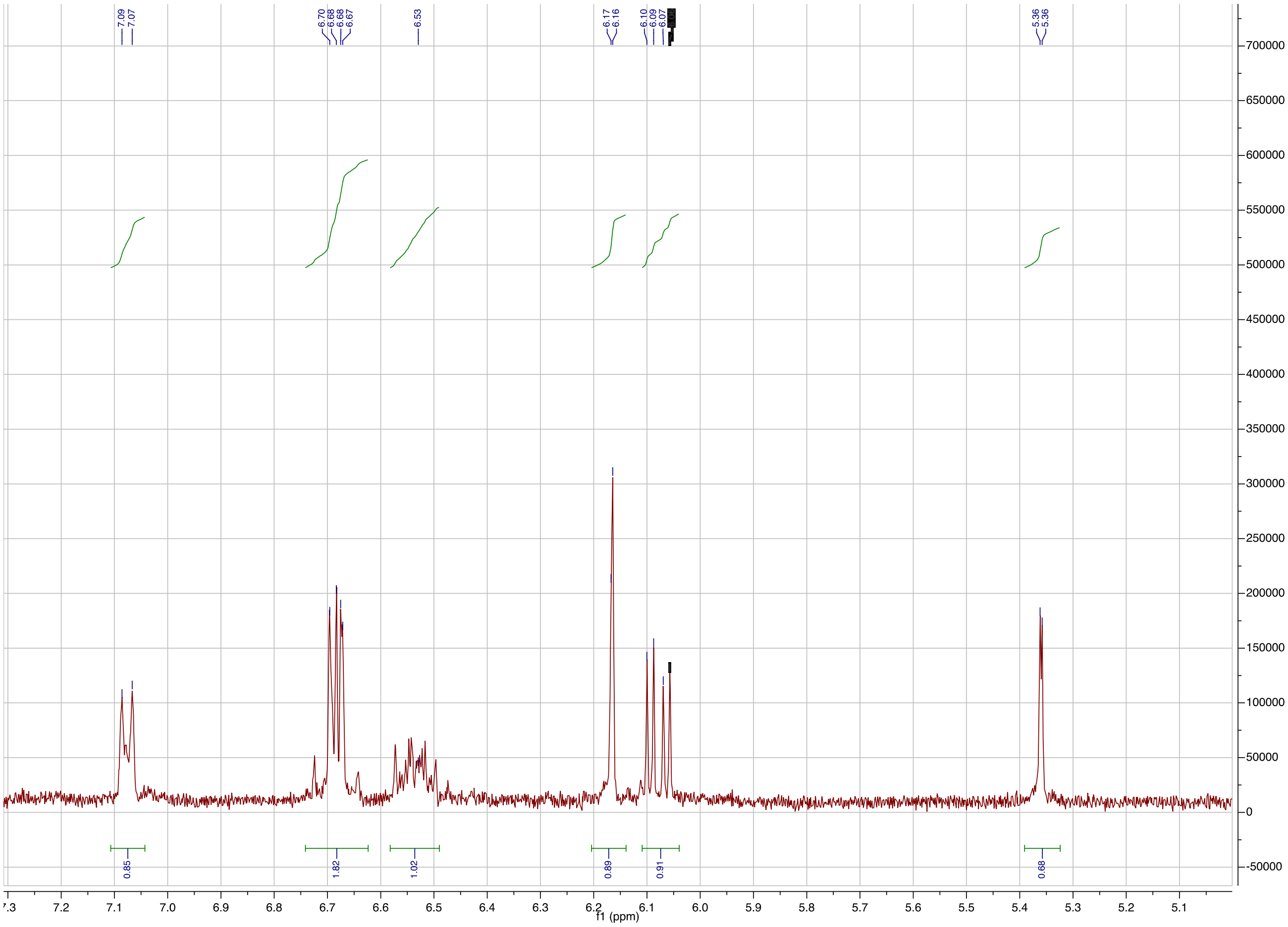

Figure S1D. ^{1}H NMR of **6**.

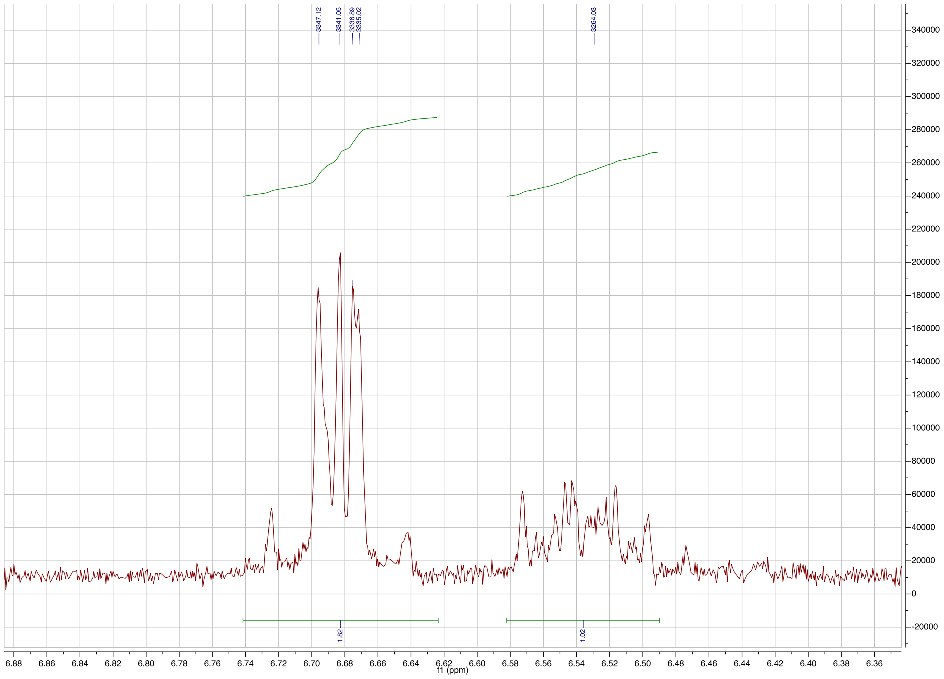

Figure S1E. ^{1}H NMR of **6**.

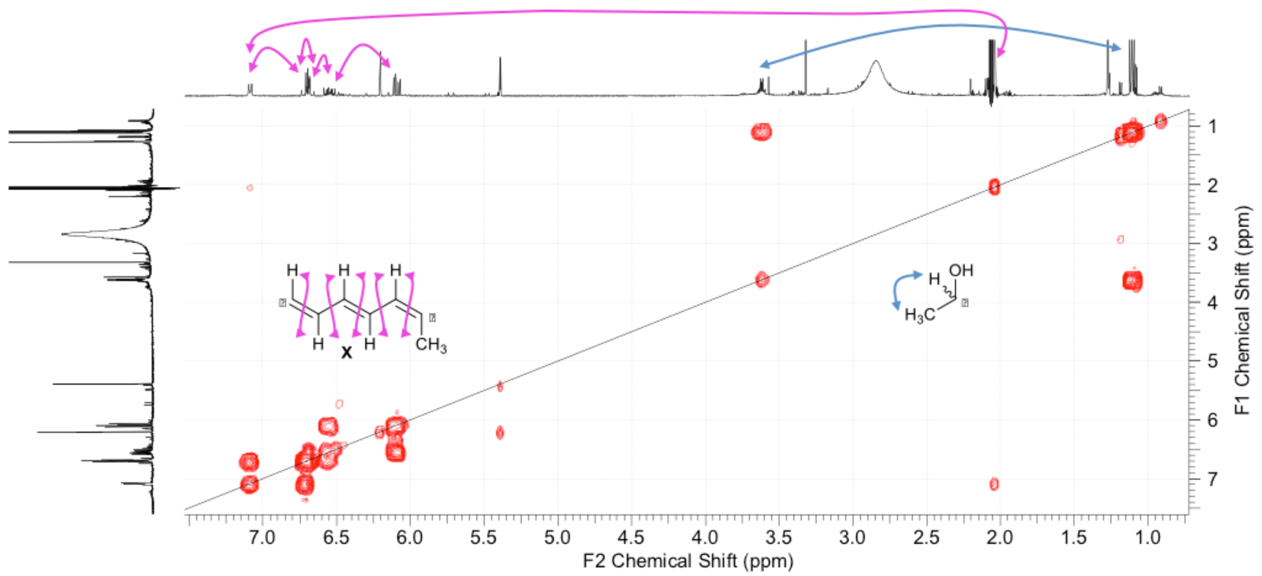

Figure S1F. ^{1}H COSY NMR of **6**.

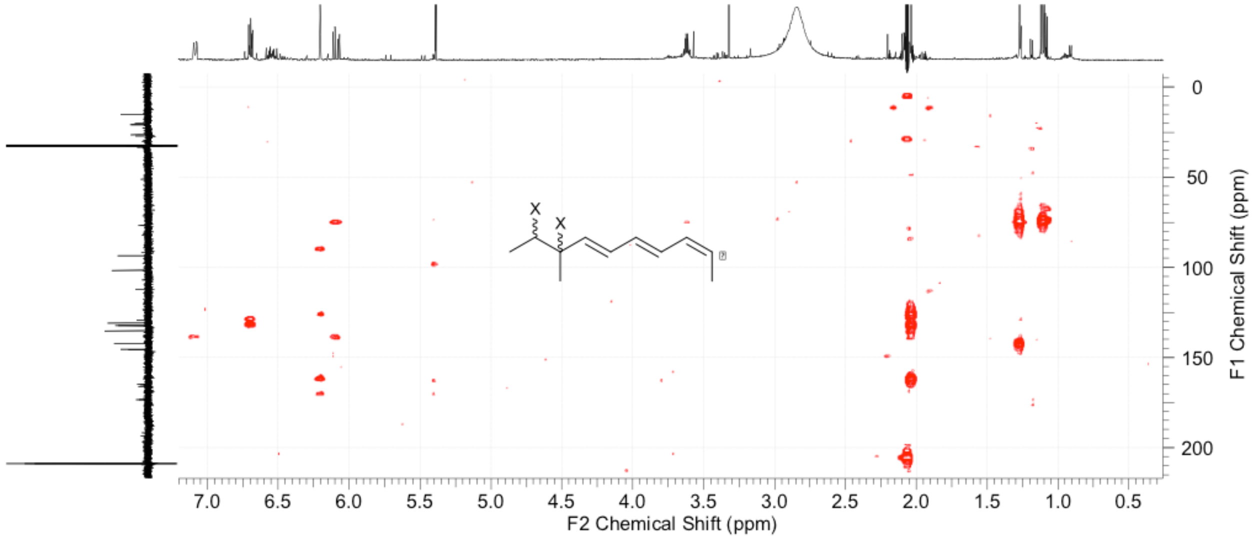

Figure S1G. HSQC NMR of **6**.

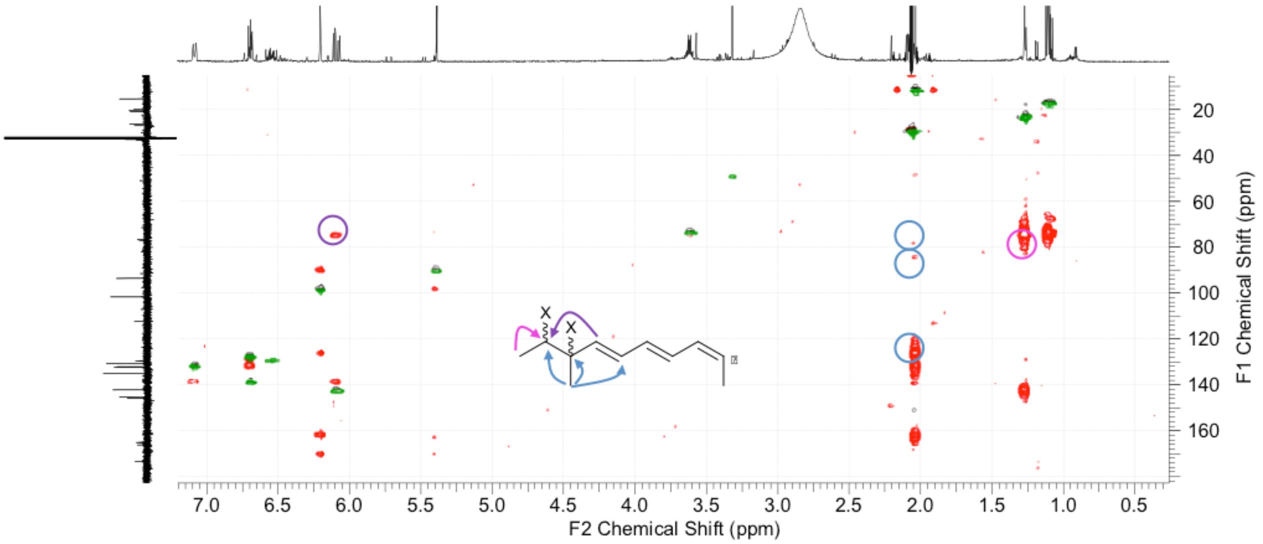

Figure S1H. HMBC NMR of **6**.

Acetate labelling

A culture of *A. oryzae* pTAex3ACE1eGFP was supplemented with sodium [1,2-$^{13}C_2$] acetate, 2 and 3 days after inoculation to a final culture concentration of ~ 6 mM. After incubation for 5 days 12,13-dihydroxymagnaporthepyrone **6** (10 mg) was purified and analysed by ^{13}C NMR. The resulting spectrum was compared to a spectrum of unlabelled 12,13-dihydroxymagnaporthepyrone **6** (Figure S1).

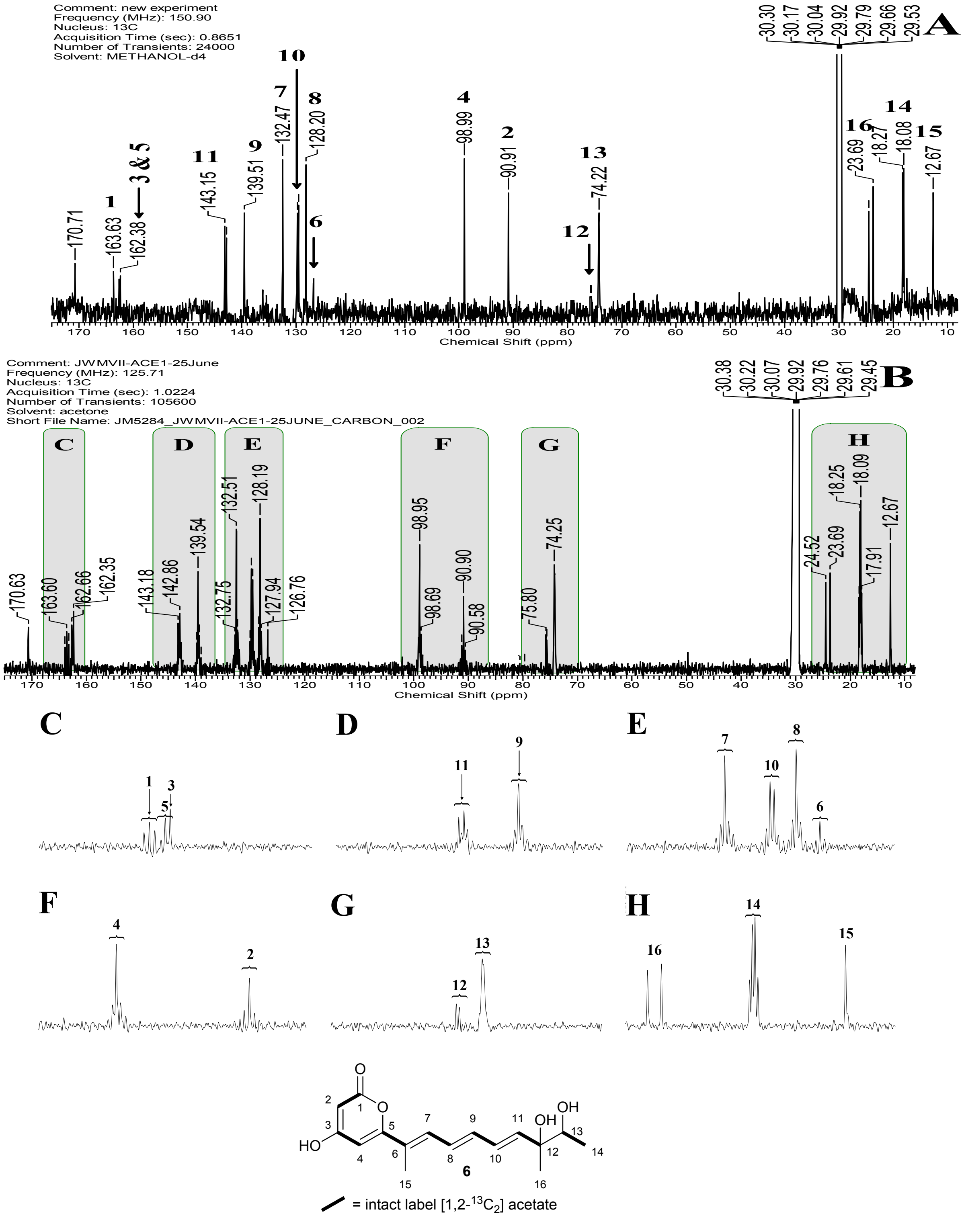

Figure S1I: ^{13}C NMR of 12,13-dihydroxymagnaporthepyrone **6**. B: ^{13}C NMR of 12,13-dihydroxymagnaporthepyrone **6** from [1,2-$^{13}C_2$] acetate labelling study with expansions (C-H)

Analysis of the ^{13}C NMR spectrum of the 12,13-dihydroxymagnaporthepyrone **6** isolated in the [1,2-$^{13}C_2$] acetate isotopic labelling study revealed evidence for intact isotopic enrichment at all positions in the carbon backbone with the exception of the two side chain methyl groups (C-15 and C-16).

The resonance corresponding to C-3 was very weak which meant no satellites could be observed, however the coupled partner C-4 shows coupling to C-3 (coupling constant analysis rules out coupling to C-5 which shows a coupling to C-6). Signals corresponding to C-12 and C-13 were both weak and complicated by the mixture of diastereoisomers. Broad peaks appear coalesced with the satellites in both cases. The coupling constants for peaks corresponding to incorporation at C-12 and C-13 could not be extracted, but the effect of coupling was evident on each respective coupling partner (C-11 & C-14). The labelling pattern observed in 12,13-dihydroxymagnaporthepyrone **6** following supplementation with [1,2-$^{13}C_2$] acetate, shows that the carbon skeleton of 12,13-dihydroxymagnaporthepyrone **6** is entirely derived from acetate.

Methionine labelling of 6.

A seed culture of *A. oryzae* pTAex3ACE1eGFP in three 500 ml flasks (containing 100 ml medium in each flask) was incubated for 3 days (72 h) in medium containing glucose (CD + 1% polypeptone + 2% glucose). The medium was removed and replaced with production medium containing starch (CD + 1% polypeptone + 2% starch). 45 mg [methyl-^{13}C]-methionine was dissolved in 3 ml sterile water and filtered through 0.2 μm syringe filter device for sterilisation. 0.5 ml of the solution was added to each flask respectively at the 48 h and 72 h fermentation (final concentration of [methyl-^{13}C] methionine to each flask was 1 mM). The culture was harvested after 96 h fermentation. The culture filtrate was extracted with ethyl acetate twice. The target compound was then purified by LCMS (< 0.5 mg).

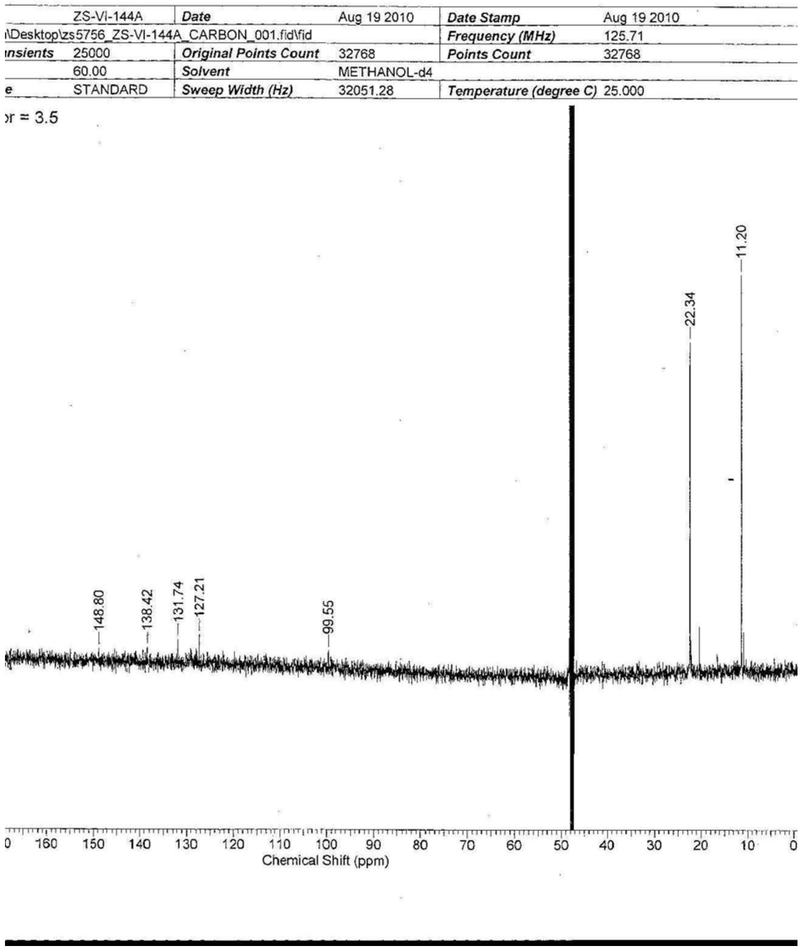

Figure S2A. ^{13}C NMR spectrum of **6** labelled with [methyl-^{13}C]-methionine. Note that NMR solvent is CD_3OD.

10,11-Dihydroxymagnaporthepyrone 7. Isolated 8 mg by preparative HPLC. ^1H NMR (500 MHz Acetone-d$_6$): □ 1.60 (3H, d, $^3J_{HH}$ 7.2, H-14), 1.65 (3H, s, H-16), 2.00 (3H, s, H-15), 3.93 (1H, d, $^3J_{HH}$ 7.2, H-11), 4.25 (1H, dd, $^3J_{HH}$ 7.7, $^3J_{HH}$ 7.7, H-10), 5.36 (1H, d, $^4J_{HH}$ 2.0, , H-2), 5.50 (1H, q, $^3J_{HH}$ 7.7, H-13), 6.16 (1H, d, $^4J_{HH}$ 2.0, H-4), 6.30 (1H, dd, $^3J_{HH}$ 15.7, $^3J_{HH}$ 15.7, H-9), 6.76 (1H, dd, $^3J_{HH}$ 15.2, $^3J_{HH}$ 15.4, H-8), 7.02 (1H, d, $^3J_{HH}$ 15, H-7); ^{13}C NMR (151 MHz Acetone-d$_6$): □ 12.1 ppm (C-14), 12.8 (C-16), 13.2 (C-15), 73.7 (C-10), 81.0 (C-11), 90.8 (C-2), 99.0 (C-4), 122.1 (C-13), 126.3 (C-6), 126.3 (C-8), 132.2 (C-7), 136.8 (C-12), 142.0 (C-9), 162.0 (C-5), 163.6 (C-1), 170.8 (C-3); UV/vis □$_{max}$ (H_2O/MeOH) 248, 340 nm; MS (ESI$^+$) *m/z* (%): 293 (100) [M]H$^+$, 315 (50) [M]Na$^+$; MS (ESI$^-$) *m/z* (%): 291 (100) [M - H]$^-$; HRMS (ESI$^-$)

calculated [M - H]$^-$ 291.1238; found 291.1238. HRMS (ESI$^+$) calculated [M]Na$^+$ 315.1203; found 315.1204.

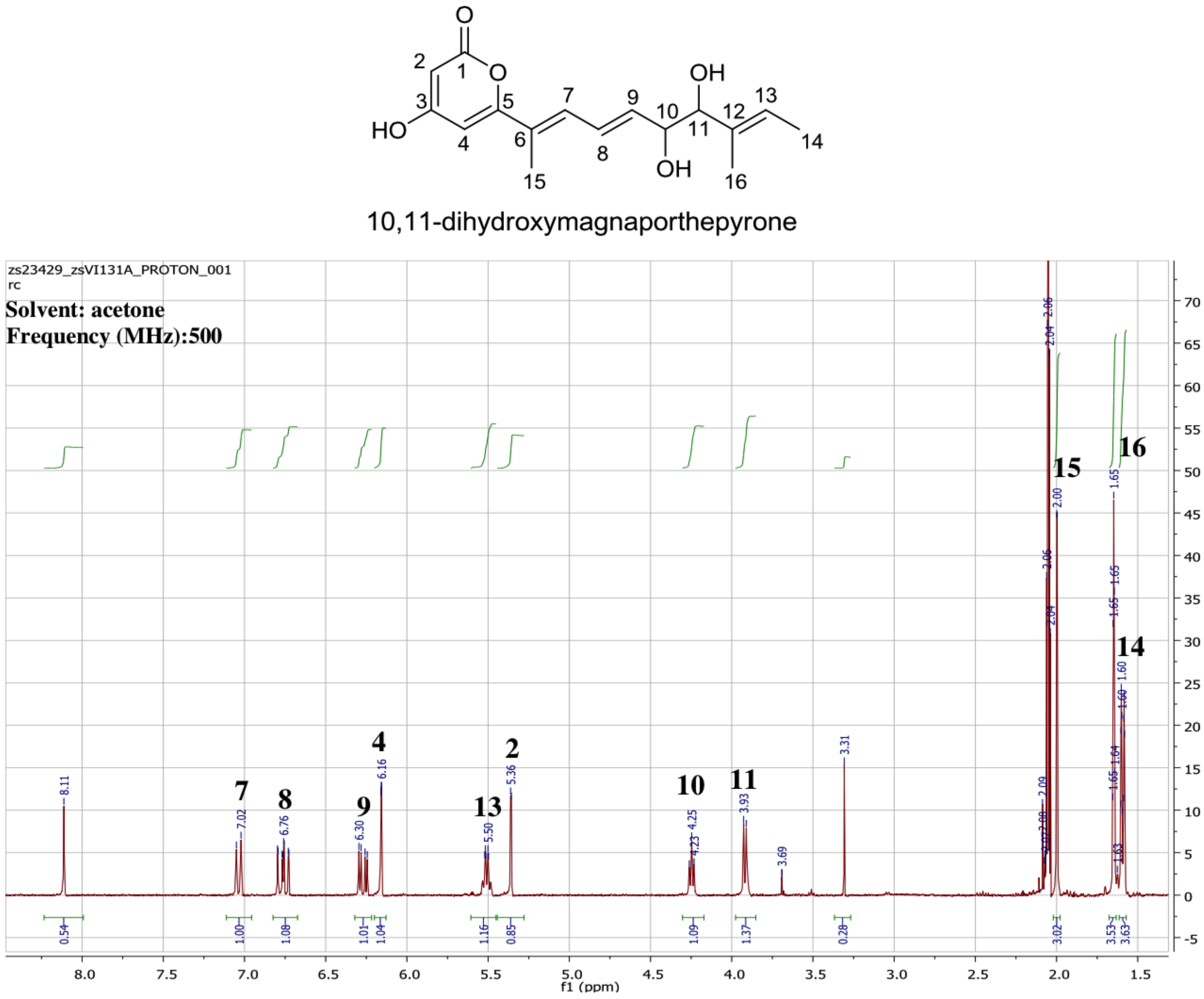

Figure S3A. ^{1}H NMR spectrum of **7**

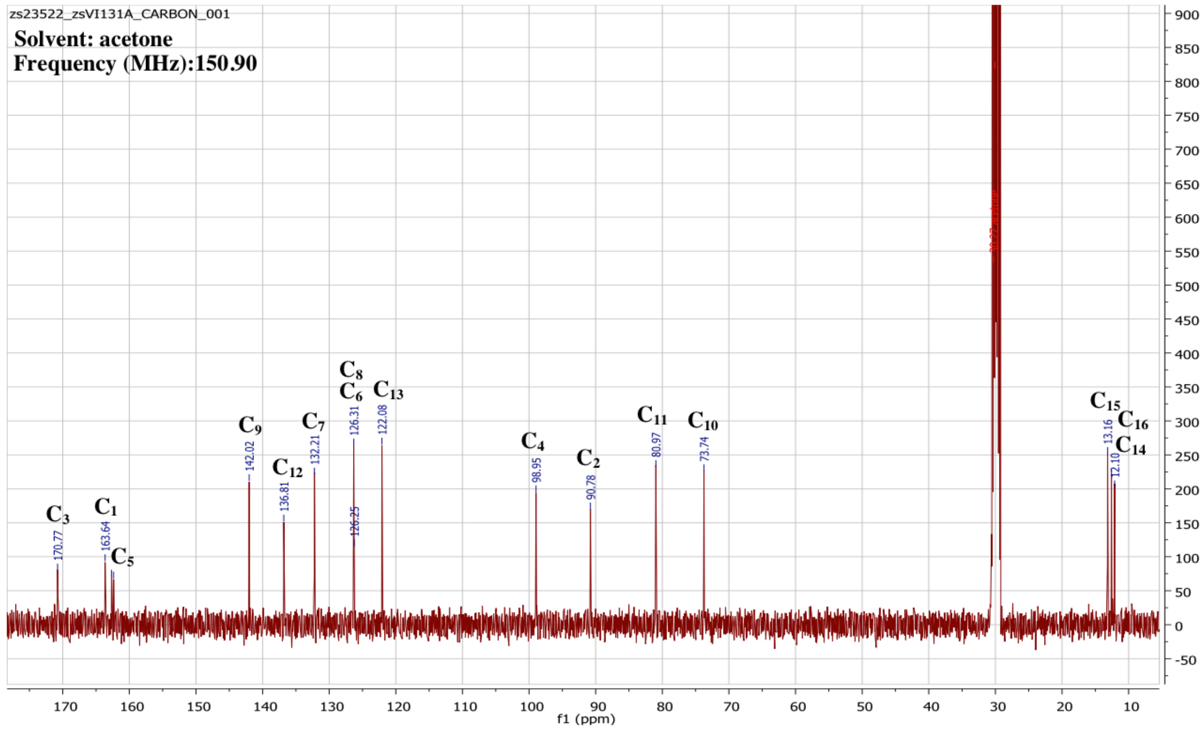

Figure S3B. ^{13}C NMR spectrum of **7**.

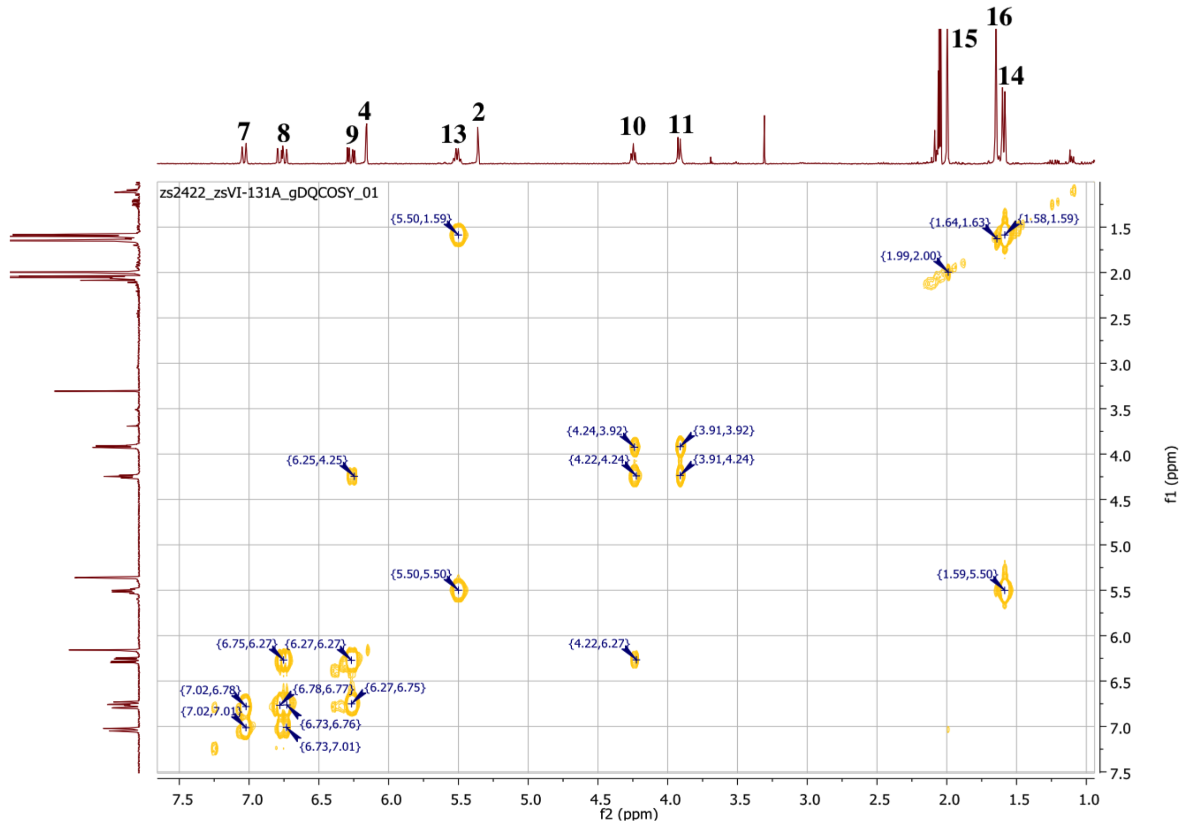

Figure S3C. COSY NMR Spectrum of **7**.

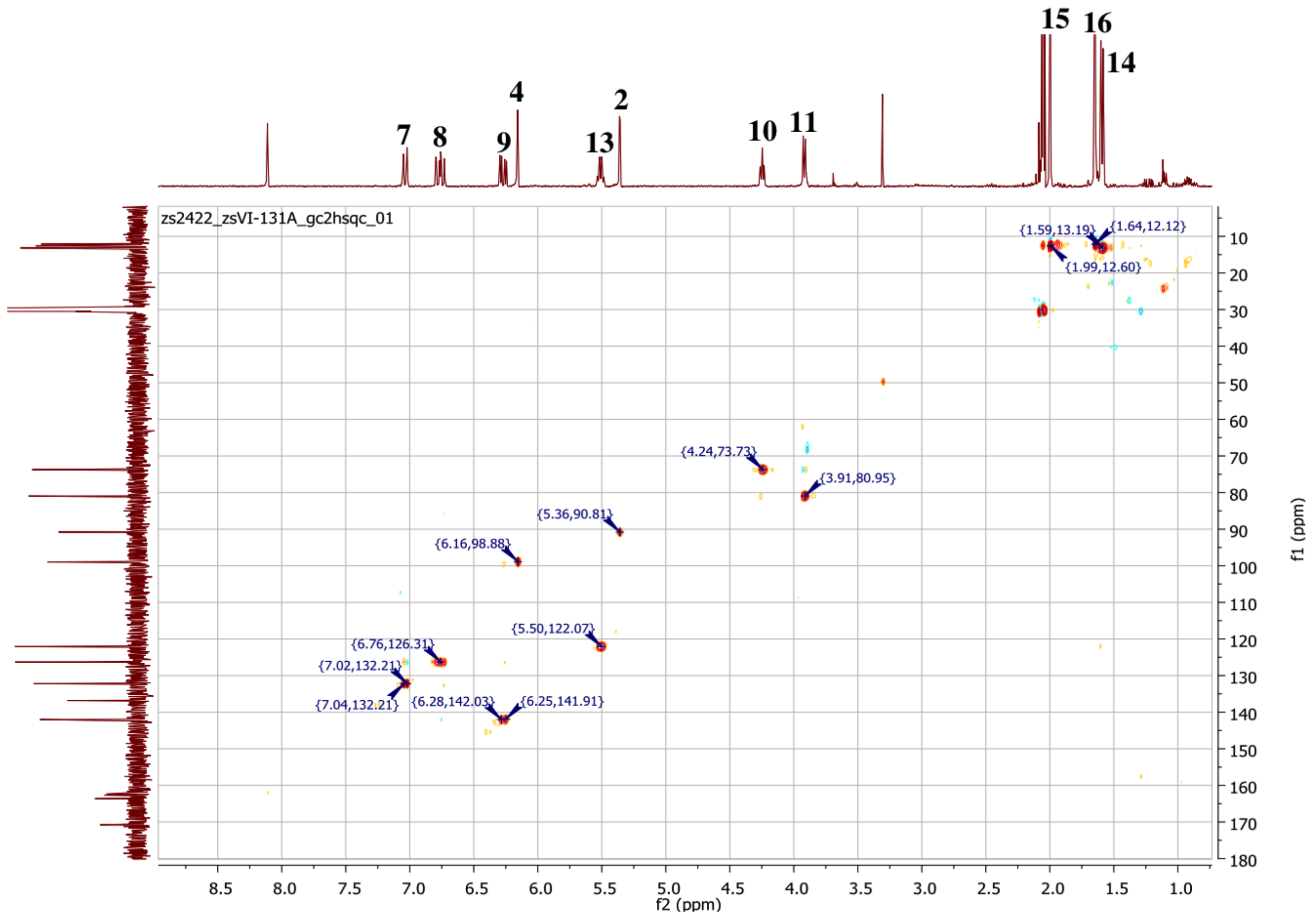

Figure S3D. HSQC NMR spectrum of **7**.

Amide 8. ^{1}H NMR (500 MHz, $CDCl_3$): □ 0.92 ppm (d, 3H, *J* = 6.8), 1.35 (m, 2H), 1.51 (m, 2H), 1.56 (s, 3H), 1.72 (d, 3H, *J* = 9.1), 1.74 (s, 3H), 1.95, (t, 2H, *J* = 7.5), 2.03 (t, 2H, *J* = 7.1), 2.41 (m, 1H), 2.47 (m, 2H), 2.76 (dd, 1H, *J* = 7.4, 13.9), 2.81 (dd, 1H, *J* = 7.1, 13.9), 3.31 (d, 1H, *J* = 17.2), 3.34 (d, 1H, *J* = 17.2), 3.57 (m, 1H), 3.68 (m, 1H), 4.15 (m, 1H), 4.91 (d, 1H, *J* = 9.3), 5.52 (q, 1H, *J* = 6.8), 5.61 (ddd, 1H, *J* = 14.6, 7.3, 7.3), 6.05 (dd, 1H, *J* = 14.3, 9.3), 6.08 (dd, 1H, *J* = 14.6, 9.3), 6.13 (d, 1H, *J* = 14.6), 6.75 (brd, 2H, *J* = 8) ,7.06 (brd, 2H, *J* = 8), 7.2 (brd, 1H, J = 7.8); ^{13}C NMR (125 MHz, $CDCl_3$): □□14.6 ppm, 16.6, 18.8, 23.4, 25.4, 29.8, 35.4, 38.8, 41.9, 43.6, 46.4, 51.2, 56.1, 67, 118.1, 128.9, 129.2, 132.0, 133, 133.7, 134.6, 134.8, 135.8, 137.4, 138.1, 157.2, 169, 209.7. HRMS ESI [M]H^+: found 482.32649; predicted for $C_{30}H_{4}3NO_{4}$ 482.32703.

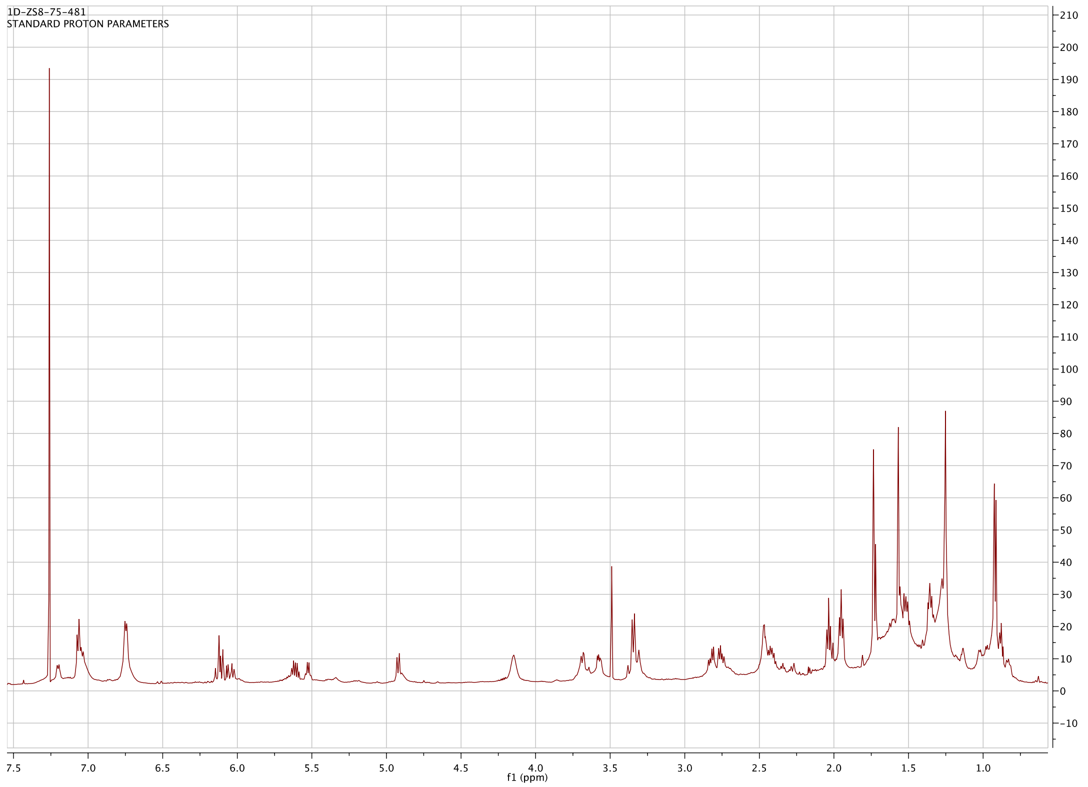

Figure S4A. ^{1}H NMR spectrum of **8**.

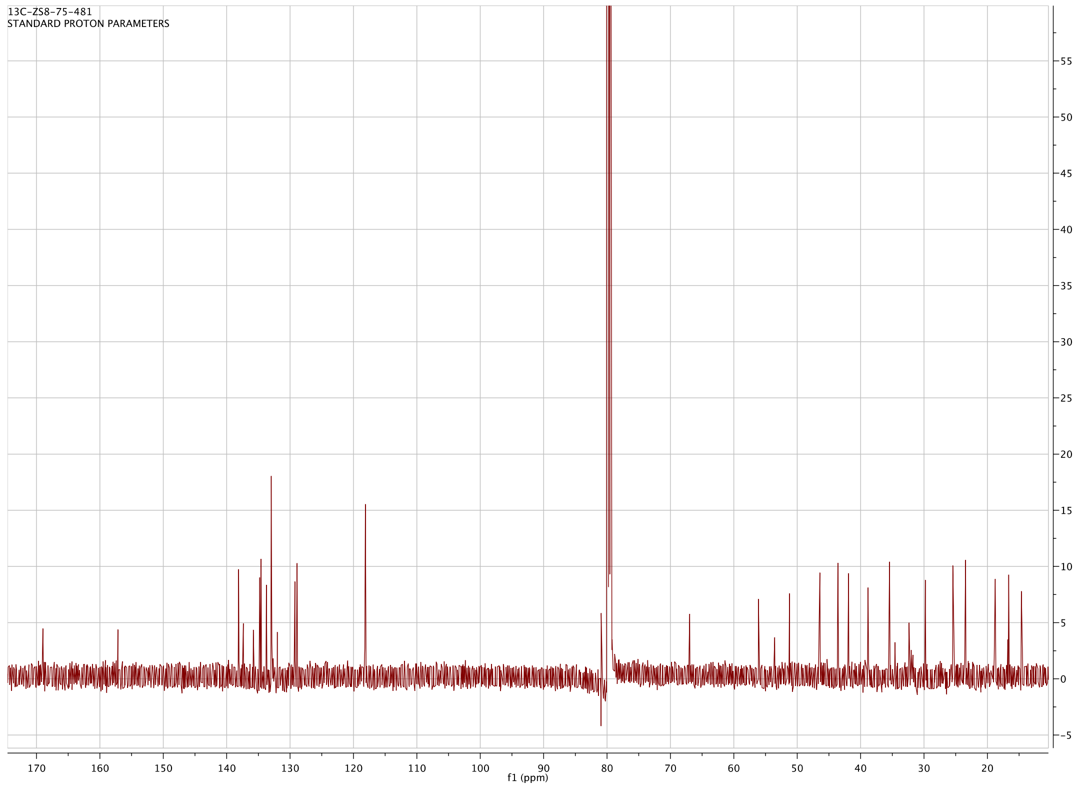

Figure S4B. ^{13}C NMR spectrum of **8**.

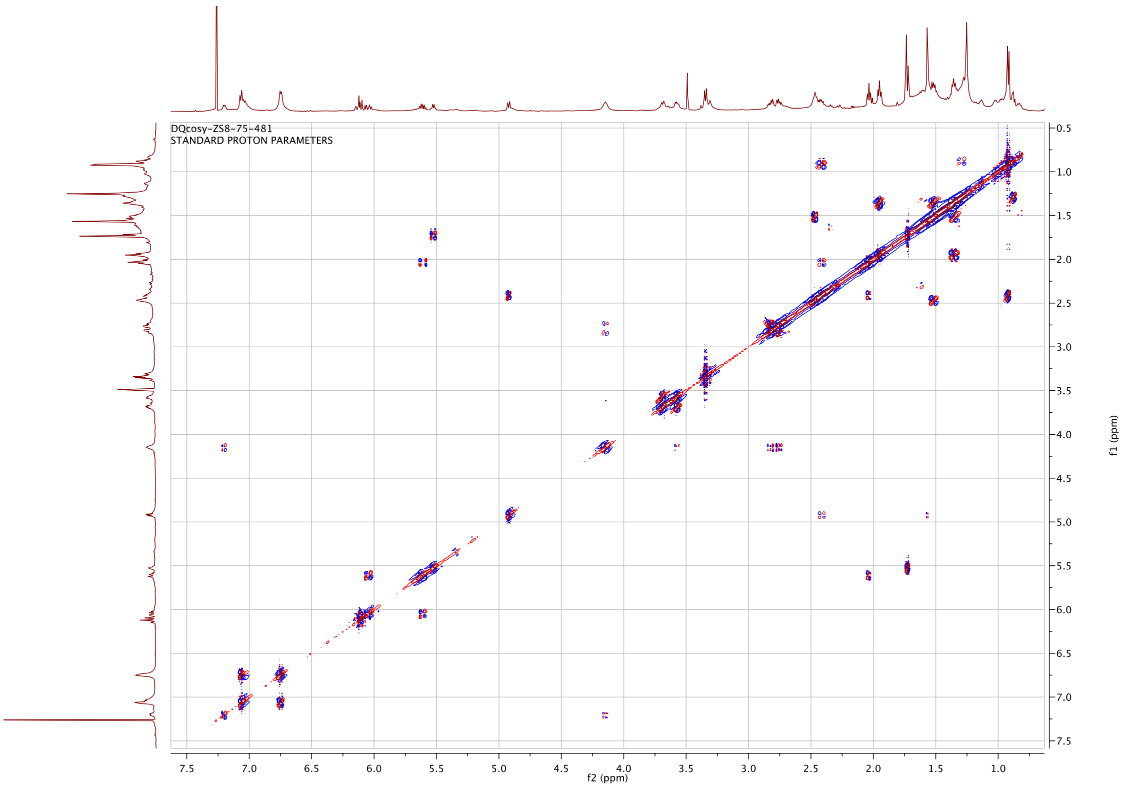

Figure S4C. COSY spectrum of **8**.

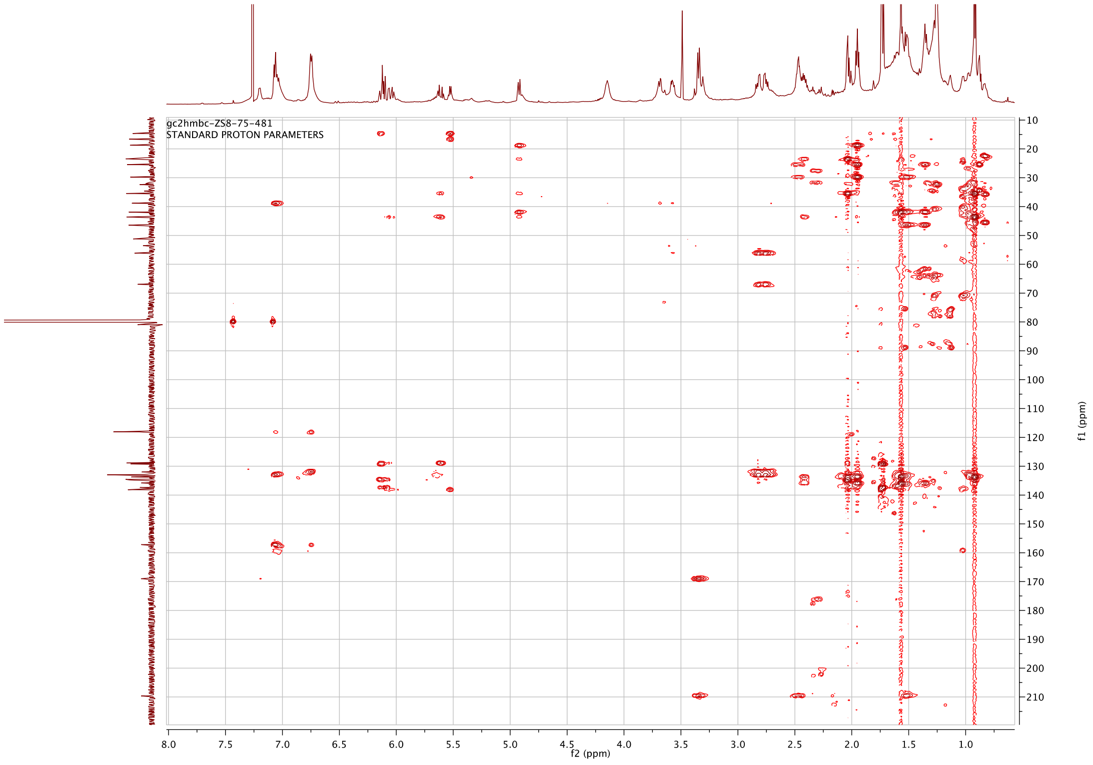

Figure S4D. HMBC spectrum of **8**.

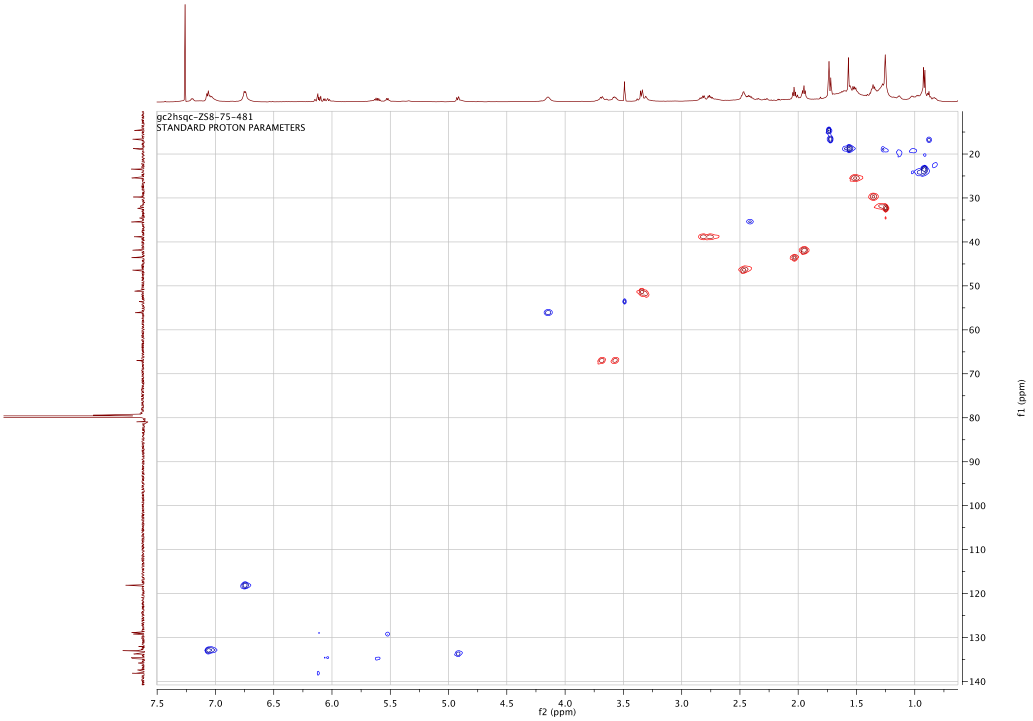

Figure S4E. HSQC spectrum of **8**.

Summary Table 2D NMR Data for 8

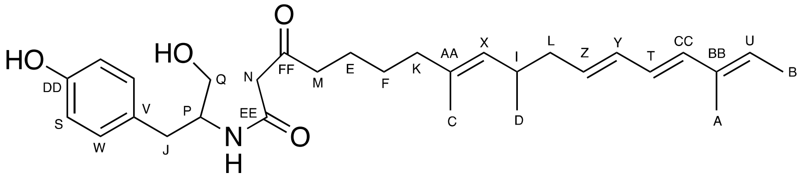

Chemical Formula: $C_{30}H_{43}NO_4$
Exact Mass: 481.31921

Position	□C	□H	m	*J*/Hz	HSQC	HMBC H to	COSY
A	14.6	1.74	s		CH3	U, BB, CC	-
B	16.6	1.72	d	9.1	CH3	BB, U	U
C	18.8	1.56	s		CH3	X, AA, K	-
D	23.4	0.92	d	6.8	CH3	I, L, X	I
E	25.4	1.51	m		CH2	F, K, M, FF	M, F
F	29.8	1.35	m		CH2	E, K, M, AA	E, K
I	35.4	2.41	m		CH	D, L, X, AA, Z	D, L, X
J	38.8	2.76, 2.81	dd, dd	7.4, 13.9 - 7.1, 13.9	CH2	P, Q, W	P
K	41.9	1.95	t	7.5	CH2	C, E, F, X, AA	F
L	43.6	2.03	t	7.1	CH2	D, I, Y, Z, X	I, Z
M	46.4	2.47	m		CH2	E, F, FF,	E
N	51.2	3.31, 3.34	d, d	17.2, 17.2		EE, FF	N geminal
P	56.1	4.15	m		CH	J	J, Q, NH
Q	67	3.57, 3.68	m, m		CH2	J, P	Q geminal, P
S	118.1	6.75	brd	8	2 x CH	S, V, DD	W
T	128.9	6.08	dd	14.6, 9.3	CH	CC, BB, Y, Z	Y, CC
U	129.2	5.52	q	6.8	CH	A, B, CC	B
V	132	-	-	-	Q	-	-
W	133	7.06	brd	8	2 x CH	J, DD	S
X	133.7	4.91	d	9.3	CH	C, D, I, K, L,	I
Y	134.6	6.05	dd	14.3, 9.3	CH	T, CC, L	T, Z
Z	134.8	5.61	ddd	14.6, 7.3, 7.3	CH	I, L, T	Y, L
AA	135.8	-	-	-	Q	-	-
BB	137.4	-	-	-	Q	-	-
CC	138.1	6.13	d	14.6	CH	A, T, U, Y, Z	T
DD	157.2	-	-	-	Q	-	-
EE	169	-	-	-	Q	-	-
FF	209.7	-	-	-	Q	-	-
NH	-	7.2	brd	7.8	NH	EE	P

5. Biological Testing of 6 - 8.

Rice plants of cultivars with (C101LAC, IR64) or without resistance gene *Pi33* (CO39, Maratelli, Sariceltik), were grown in pots placed in a greenhouse for four weeks (4-5 leaf stage), as previously described[3]. The last fully developed rice leaf was attached to a horizontal board and wounded by pressing with a pipet tip at four locations. A 10 µL droplet of a metabolite or control solution was deposited on the wounded area. In some experiments, a drop of 15 µL corresponding to a spore suspension of a *M. oryzae* isolate virulent on *Pi33* (PH19, see reference 3) was added to the wounded site, after evaporation of the metabolite droplet (1-3 hours). The cover of a Petri dish was placed on each leaf and plants were placed in a dew chamber overnight at 22 °C and saturating humidity. Plants were then moved back to the greenhouse and symptoms were observed from the first day after inoculation up to 7 days. Compounds were dissolved in Methanol (MeOH) at 10^{-1} M and diluted in water to 10^{-2} and 10^{-3} M, reaching final methanol concentrations of 100, 10 and 1%, respectively. Each metabolite and control solutions (MeOH, water) were deposited at 4 locations on 4 rice leaves (16 points). The experiment was repeated twice independently.

6. Reverse-Transcription Analysis of ACE1 Introns

The RT-PCR products obtained for each of the three *ACE1* introns were cloned into pGEM-T-Easy Vector and sequenced. The resulting sequences were aligned (Figure S5).

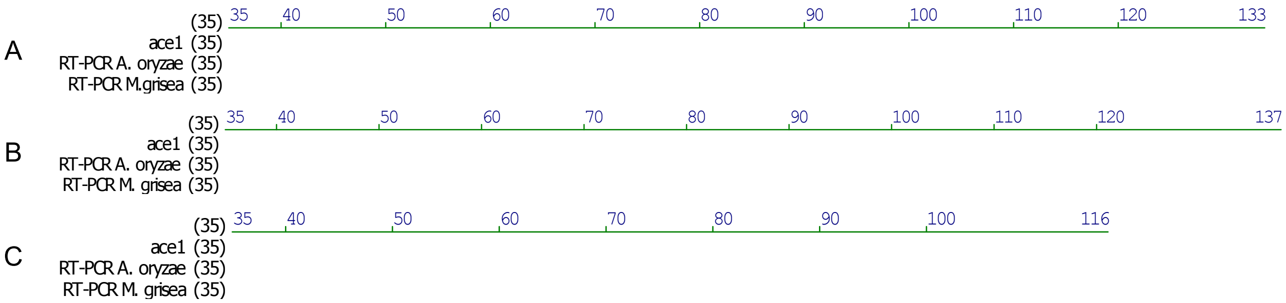

Figure S5. Alignment of sequencing results after RT-PCR. **A**, across intron-1 splicing area following expression in *A. oryzae* and *M. oryzae*; **B**, across intron-2 splicing area following expression in *A. oryzae* and *M. oryzae*; **C**, across intron-3 area following expression in *A. oryzae* and *M. oryzae*.

7. Bioinformatic Analysis and Discussion of the *ACE1* C-terminal domain.

The terminal R domain of PKS-NRPS enzymes are known to offload the polyketide-amino acid intermediate in two possible ways; via a Dieckmann cyclisation (DKC) to generate a tetramic acid moiety[6] or *via* a reductive release.[7] Walsh and co-workers studied the α-cyclopiazonic acid synthase (CpaS) R* domain from *Aspergillus* sp., by heterologous overproduction of the terminal domains in *E. coli* and determined that the R* domain catalyses a Dieckmann type cyclisation and does so without the participation of NAD(P)H, concluding that R* domains do not function as reductases.[8] They further investigated the CpaS R* domain to identify residues that contribute to its redox-independent cyclase activities and observed that mutations in the Ser-Tyr-Lys catalytic triad, notably Tyr to Phe / Leu, abolish the reductase activity.

Characterization of the R domain of equisetin synthase (EqiS) appeared to contradict Walsh and co-workers' results. The EqiS R domain has a conserved Ser-Tyr-Lys catalytic triad which is expected for reductive domains, however over-expression of the R domain in *E. coli* determined that the R domain did not perform a reducing function nor did it bind reducing co-factors.[9] Recently it has emerged that EqiS is not the PKS-NRPS responsible for equisetin biosynthesis, but is the PKS-NRPS responsible for fusaridione A biosynthesis and has been reassigned as fsdS.[10] Therefore, the exact nature of the equisetin synthase (renamed eqxS) R domain is currently unknown. The initial experiments investigating eqiS (now fsdS) used synthetic precursors based on the structure of *equisetin* substrate mimics and not fusaridione substrate mimics, perhaps questioning the validity of the DKC activity assignment. Although *fsdS* has been over-expressed in its native host *Fusarium heterosporum* and did appear to function as a DKC to produce fusaridione A, the compound was unstable and degrades via a reverse-Dieckmann reaction to open the pyrrolidinedione ring. Since *fsdS* was expressed in its native host there is a possibility that another enzyme was active at the same time, facilitating the Dieckmann cyclisation. Therefore, the exact functions of the R domains from eqxS and fsdS remain ambiguous.

A multiple sequence alignment of several characterized PKS-NRPS R domains (Figure S6) appears to distinguish R* (DKC) domains from reducing (R) domains based on the Tyr to Phe / Leu mutation in the catalytic triad. Tenellin synthase (TENS), cyclopiazonic acid synthase (CpaS), desmethylbassianin synthase (DmbS) and aspyridone synthase (ApdA) are known to arise via a Dieckmann cyclisation and all have either a Phe or Leu mutation in the Ser-Tyr-Lys catalytic triad. ACE1, fusarin synthase (FusS), cytochalasin synthase (ccsA), chaetoglobosin synthase (CheS), pseurotin synthase (PsoA) and the uncharacterized synthase SYN2 are assigned as reductive domains

based on the conserved Ser-Tyr-Lys motif. Experimental evidence from heterologous expression confirms that ACE1 and ccsA[11] use a reductive release and experimental evidence from gene knock outs in the fusarin gene cluster also suggests a reductive release.[7] The R domain activity of CheS and PsoA has not yet been confirmed to the best of our knowledge. The eqxS and fsdS R domains are also assigned as reducing domains based on the alignment and since the experimental evidence reported does not fully disprove this, and it is out of the scope of the project to investigate them further, the assignment appears valid for the time being.

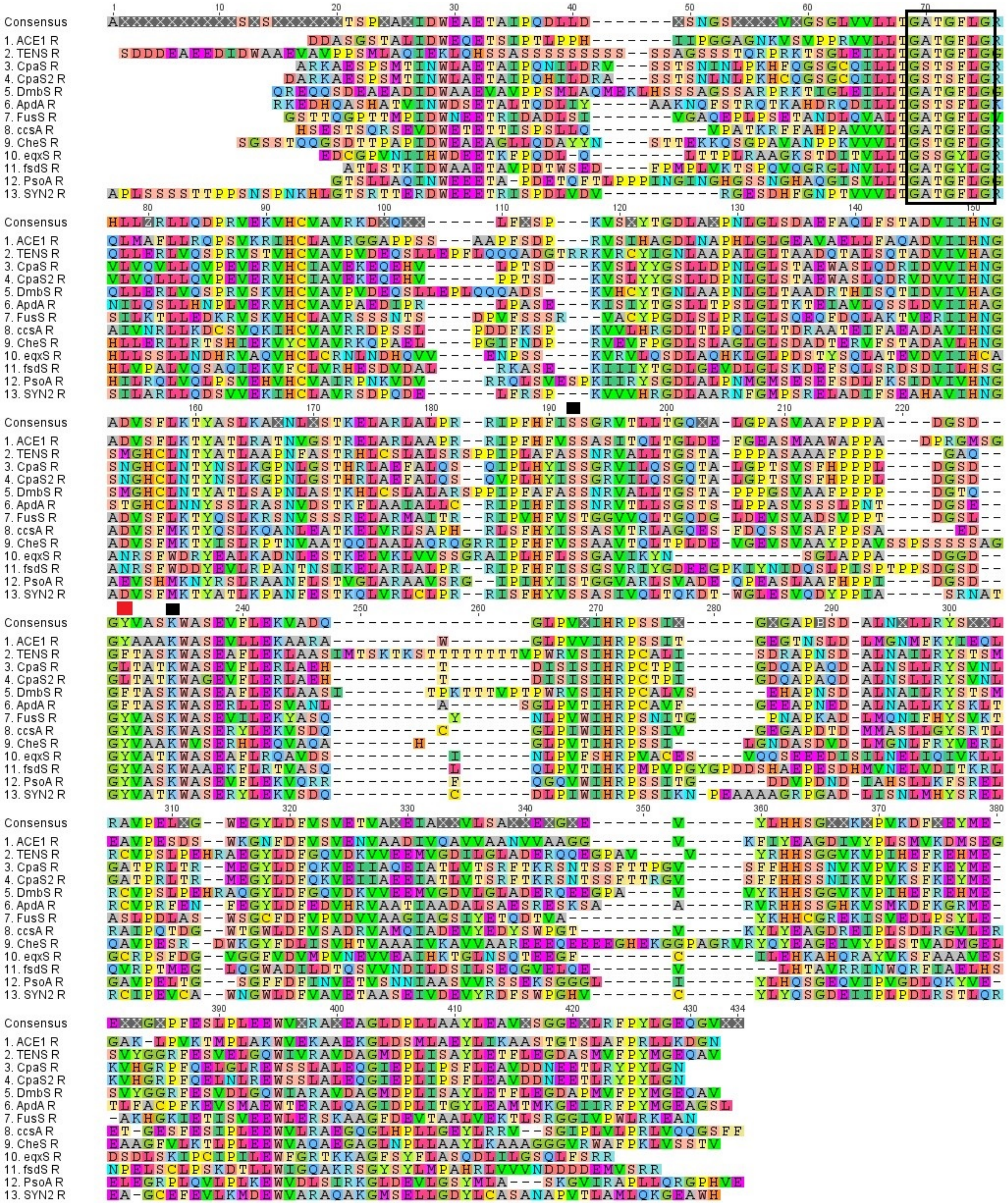

Figure S6. Sequence alignment of the C-terminal domain from fungal PKS-NRPS utilizing both the DKC mode of release (TENS, CpaS, CpaS2, DmbS and ApdA) and a predicted reductive release (ACE1, FusS, ccsA, CheS, eqxS, fsdSPsoA and SYN2). Sequences were obtained from NCBI and submitted to antiSMASH[4] to identify domain boundaries. The resultant C-terminal domains were aligned using Geneious.[5] The black box indicates the conserved NADH/NADPH binding domain; the solid squares indicate the SDR Ser-Tyr-Lys catalytic triad; the red square indicates the Tyr -> Phe / Leu mutation which abolishes the reductase activities. R represents the abbreviation of reductase. Protein name abbreviations: TENS (tenellin synthase), CpaS (cyclopiazonic acid synthase, *A. flavus* NRRL3357), CpaS 2 (cyclopiazonic acid synthase, *A. oryzae* NBRC4177), DmbS (desmethylbassianin synthase), ApdA (aspyridone synthase), FusS (fusarin synthase), ccsA (cytochalasin synthase, ACLA_078660, CheS (chaetoglobosin synthase, CHGG_01239), eqxS (equisetin synthase), fsdS (fusaridione synthase), PsoA (pseurotin synthase), SYN2 (uncharacterised synthase).

8. Comparative analysis of the ACE1, CHGG1239 and *ccs* PKS-NRPS gene clusters

ACE1 gene cluster from Magnaporthe oryzae		***CHGG1239* gene cluster from *Chaetomium globosum***			***ccs* gene cluster from *Aspergillus clavatus* NRRL 1**		
**Assigned gene name/ Gene locus (MGG_)**	***Deduced function***	**Assigned gene name / Gene locus (CHGG_)**	**Deduced function**	**Identity to ACE1 homologs***	**Assigned gene name/ Gene locus (ACLA_)**	**Deduced function**	**Identity to ACE1 homologs***
ACE1 12447	PKS-NRPS hybrid	01239	PKS-NRPS hybrid	***47%***	***ccsA*** 078660	PKS-NRPS hybrid	45%
RAP1 08391	Enoyl reductase	01240	Enoyl reductase	***62%***	***ccsC*** 078700	Enoyl reductase	46%
ORFZ 08390	Esterase	01246	Esterase	***69%***	***ccsE*** 078680	Esterase	58%
OXR1 08389	dehydrogenase	01245	dehydrogenase	***50%***	n/a	n/a	n/a
CYP1 08387	P450 monooxygenase	01243	P450 monooxygenase	***59%***	***ccsD*** 078670	P450 expoxidase	43%
BC2 08386	Zn(II)2Cys6 transcription factor	01237	Zn(II)2Cys6 transcription factor	***22%***	***ccsR*** 078640	Transcription activator	11%
OXR2 15927	FAD oxidase	01242-2	FAD oxidase	***46%***	n/a	n/a	n/a
CYP2 15928	P450 monooxygenase	01242-1	P450 monooxygenase	***37%***	***ccsG*** 078710	P450 monooxygenase	37%
MFS1 08384	MFS transporter	n/a	n/a	n/a	n/a	n/a	n/a
ORF3 08381	Unknown	01241	Unknown	***46%***	***ccsF*** 078690	Unknown	64%

Table S2 A comparison of the *ACE1* gene cluster with the characterized gene clusters for chaetoglobosin (*CHGG1239* gene cluster from *Chaetomium globosum*) and cytochalasin (*ccs* gene cluster from *Aspergillus clavatus* NRRL 1) biosynthesis. * Percentage values represent protein identity to ACE1 cluster homologs.

9. References

1. K. A K Pahirulzaman, K. Williams, and C. M. Lazarus, *Meth. Enzymol.*, 2012, **517**, 241–260.
2. *Communication entre les plantes et leurs agents pathogens : role des metabolites secondaires dans la reconnaissance du champignon* Magnaporthe grisea *par le riz,* J. Collemare, Ph.D. Thesis, Universite Paris XI, 2007.

3 Berruyer R, Adreit H, Milazzo J, Gaillard S, Berger A, Dioh W, Lebrun MH, Tharreau D (2003) Identification and fine mapping of *Pi33*, the rice resistance gene corresponding to the *Magnaporthe grisea* avirulence gene *ACE1*. *Theor Appl Genet*, **107:**1139–1147.

4. K. Blin, M. H. Medema, D. Kazempour, M. A. Fischbach. R. Breitling, E. Takano and T. Weber, *Nucl. Acids Res.*, 2013, **41**, W1, W204 – W212
5. Drummond A.J., *et al.*, 2010, Geneious v7.1.4. Available at http://www.geneious.com
6. Halo L. M., Marshall J. W., Yakasai A. A., Song Z., Butts C. P., Crump M. P. Heneghan M., Bailey A. M, Simpson T. J., Lazarus C. M. and Cox R. J., *ChemBioChem*, 2008, **9**, 585-594
7. Niehaus E-M., Kleigrewe K., Wiemann P., Studt L., Sieber C. M. K., Connolly L. R., Freitag M., Güldener U., Tudzynski B., Humpf H-U., *Chemistry and Biology*, 2013, **20**, 1055-1066
8. X. Liu and C. T. Walsh, *Biochemistry*, 2009, **48**, 8746-8757
9. J. W. Sims and E. W. Schmidt, *J. Am. Chem. Soc.*, 2008, **130**, 11149-11155
10. T. B. Kakule, D. Sardar, Z. Lin and E. W. Schmidt, *ACS Chem. Biol.*, 2013, **8**, 1549-1557
11. R. Fujii, A. Minami, K. Gomi, H. Oikawa, *Tet. Lett.*, 2013, **54**, 2999 - 3002
